# Has Regulatory Action Reduced Human Exposure to Flame Retardants?

**DOI:** 10.1021/acs.est.3c02896

**Published:** 2023-11-22

**Authors:** Veronica van der Schyff, Jiří Kalina, Annalisa Abballe, Anna Laura Iamiceli, Eva Govarts, Lisa Melymuk

**Affiliations:** † RECETOX, Faculty of Science, Masaryk University, Kotlarska 2, 61137 Brno, Czech Republic; ‡ Department of Environment and Health, Italian National Institute for Health, Viale Regina Elena 299, 00161 Rome, Italy; § VITO Health, Flemish Institute for Technological Research (VITO), 2400 Mol, Belgium

**Keywords:** flame retardant, polybrominated diphenyl ether, hexabromocyclododecane, breast milk, biomonitoring, temporal trends, effectiveness evaluation

## Abstract

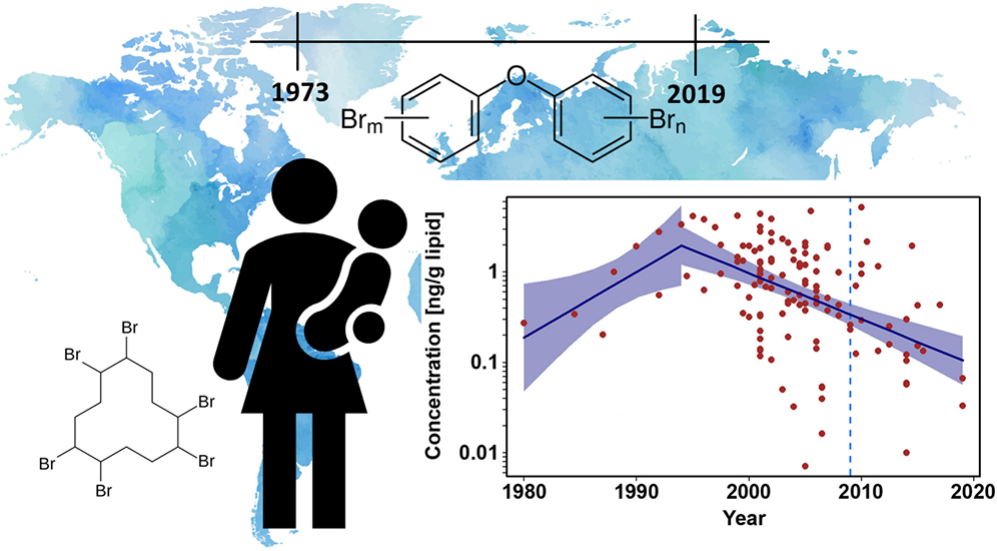

Flame retardant (FR) exposure has been linked to several environmental and human health effects. Because of this, the production and use of several FRs are regulated globally. We reviewed the available records of polybrominated diphenyl ethers (PBDEs) and hexabromocyclododecanes (HBCDDs) in human breast milk from literature to evaluate the efficacy of regulation to reduce the exposure of FRs to humans. Two-hundred and seven studies were used for analyses to determine the spatial and temporal trends of FR exposure. North America consistently had the highest concentrations of PBDEs, while Asia and Oceania dominated HBCDD exposure. BDE-49 and -99 indicated decreasing temporal trends in most regions. BDE-153, with a longer half-life than the aforementioned isomers, typically exhibited a plateau in breast milk levels. No conclusive trend could be established for HBCDD, and insufficient information was available to determine a temporal trend for BDE-209. Breakpoint analyses indicated a significant decrease in BDE-47 and -99 in Europe around the time that regulation has been implemented, suggesting a positive effect of regulation on FR exposure. However, very few studies have been conducted globally (specifically in North America) after 2013, during the time when the most recent regulations have been implemented. This meta-analysis provides insight into global trends in human exposure to PBDEs and HBCDD, but the remaining uncertainty highlights the need for ongoing evaluation and monitoring, even after a compound group is regulated.

## Abstract 2

Flame retardant levels in breast milk differ globally due to past flammability standards, and time trends suggest recent regulatory action has been effective at reducing some exposures.

## Introduction

Flame retardants are added to a wide range of consumer products and materials in order to reduce ignition or flammability of a material or fulfill fire safety requirements.^1^ However, past efforts to reduce flammability through the addition of synthetic organic flame retardants have led to negative impacts on human and environmental health due to exposure to harmful chemicals.^2^

Polybrominated diphenyl ethers (PBDEs) and hexabromocyclododecanes (HBCDDs) were among the dominant FRs used for decades.^3,4^ The three technical mixtures of PBDEs (penta-, octa-, and deca-BDE) had multiple uses, including polyurethane foam, electrical and electronic equipment, building materials, and vehicle parts.^5^ Technical HBCDD (a mixture of the stereoisomers, α-, β-, and γ-HBCDD, with γ-HBCDD being the most abundant) was primarily used in electronics, textiles, and especially in expanded (EPS) and extruded polystyrene (XPS) applied as construction and packing materials.^6,7^

PBDEs and HBCDDs are known to be persistent, bioaccumulative, and subject to long-range transport in the environment and are ubiquitous across environmental systems.^8,9^ PBDEs have been reported in human blood,^10−12^ adipose tissues,^13−15^ and milk^16−19^ since the early 1990s, and evidence of human exposure to HBCDDs arose shortly thereafter.^20−23^

PBDE exposure has been associated with numerous adverse health outcomes, including alterations to thyroid function, reproductive systems, and breast cancer,^24−26^ with strong evidence for neurodevelopmental impacts, including lower IQ and ADHD.^27,28^ Elevated levels of PBDEs in breast milk have specifically been associated with neurodevelopmental effects and alterations to the gut microbiome in young children.^29,30^ Similar adverse effects are associated with elevated HBCDD concentrations, including endocrine disruption, specifically thyroid, neurobehavioral, and developmental disorders.^26,31^

In response to concerns regarding the environmental and human health impacts of certain FRs, actions were taken to reduce production.^32^ In 2004, the European Union stated that “in order to protect health and the environment the placing on the market and the use of pentaBDE and octaBDE and the placing on the market of articles containing one or both of these substances should be prohibited”,^33^ and in the same year these mixtures were voluntarily withdrawn from the U.S. marketplace by their manufacturers.^34^ The lower brominated PBDE congeners, tetra- and penta-BDE (main components of commercial penta-BDE^4^^35^) and hexa- and hepta-BDEs (main components of commercial octa-BDE^4^)^36^ were listed in the Stockholm Convention on Persistent Organic Pollutants (POPs) in 2009, requiring parties to eliminate the production and use of the compounds. Deca-BDE, the fully brominated PBDE molecule and main component of the decaBDE commercial product,^37^ was similarly listed in 2017.^38^ In 2008, HBCDDs were recognized as substances of very high concern (SVHC) in the EU due to environmental and human health risks^39,40^ and were added to the Stockholm Convention in 2013.^41^ In specific cases, individual countries were permitted continued production of HBCDDs until 2024.^7^

The evaluation of temporal patterns of a chemical’s concentration in a predetermined medium is an effective tool to determine the efficiency of policy in mitigating chemical exposure.^42^ Recent studies have identified declines in components of the penta- and octa-BDE technical mixtures,^9^ in air (1993–2018),^43^ soil (1998–2008),^44^ sediment (2002–2012),^45^ sewage sludge (2004–2010),^46^ and fish (1980–2009),^47^ while BDE-209 has been stable or increasing in many matrices.^46,48,49^ In humans, declines in levels of less brominated PBDEs and a more recent plateau have been identified in several countries, however, this is not uniform across regions or matrices:^9,22,50,51^ there is a lack of understanding of how generalizable these regional trends are. For HBCDDs, there is even less evidence of a global trend. Although time trends of HBCDD concentrations in multiple environmental matrices^52−54^ have been determined, analysis of temporal patterns of HBCDDs has been limited to regional scales. No consensus or clear global time trend of HBCDD concentrations in humans has been identified,^9^ with some studies reporting an increase of HBCDDs,^54−56^ others a decrease^57^ or no trend.^58,59^

Studying the effectiveness of policies concerning FRs through time trend analysis of biomonitoring data presents inherent complexities. Different FRs, each with distinct chemical structures, are incorporated into diverse applications such as furniture, electronics, and building insulation, which are associated with different emission and exposure routes.^4^ After restriction, continued presence of FR-containing products further complicates this, as different product types have very different replacement rates (e.g., smartphones, 2–6 years^60^ vs building insulation, 30–50 yrs^61^). The environmental persistence of compounds is generally longer indoors^62,63^ and indoor levels are sustained until active removal of sources.^64,65^ Exposure to FRs is further influenced by regional factors like building and cleaning practices and dietary patterns.^66^ Moreover, the differing persistence of FRs within the body complicates our understanding of exposure,^67^ with some FRs possessing longer half-lives in human tissues^68^ and partitioning within body tissues varying by compound/congener.^69,70^ Concentrations of POPs in human tissues typically reflect long-term exposures, for example, variations of PCB concentrations in breast milk levels can be explained by differences in early life exposures rather than current dietary exposures.^71^ Despite the above-mentioned complexities, our insight into the effectiveness of restrictions and the remaining risks to human populations from legacy FRs can be improved by multistudy analyses to understand and interpret the global time patterns of PBDEs and HBCDDs in humans.

In this analysis, we first review available records of PBDE and HBCDD in human milk to determine time patterns of global human exposure to FRs. Second, we evaluate the impact of regulations that were introduced over the past 20 years on exposure to these legacy FRs. Finally, we investigate the regional differences in exposure to FRs in relation to use. We supplement this with a review of past studies evaluating temporal patterns of PBDEs and HBCDDs in human matrices, to provide a comprehensive review of trends in global exposure.

## Methods

### Rationalization of Study Matrix

It is impossible to select a single biological matrix that encompasses the global population, as well as all target compounds. Due to the lipophilic nature of PBDEs and HBCDDs, they are best evaluated through matrices such as blood serum or breast/maternal milk.^72^ Breast milk has a high lipid content and can be collected noninvasively, making it a reliable and accessible matrix for assessing body burdens of PBDEs and HBCDDs, as well as many other POPs. The Stockholm Convention, in cooperation with the World Health Organization, has identified human milk as a core matrix of its Global Monitoring Plan and supported with routine quantification in pooled milk samples.^70,73^

In addition to its importance in routine monitoring programs, maternal milk is an ideal matrix for meta-analyses of biomonitoring data because of the relative homogeneity of the study population: all female, with an age range generally spanning 18–45 years. Moreover, breast milk is not only an indicator of human exposure but also represents a direct exposure route to infants, and breast milk is typically the most important exposure pathway of young children to POPs, including PBDEs.^74,75^

### Search Strategy and Selection Criteria–Human Milk Meta-Analysis

A literature search of peer-reviewed studies and reports produced by regulatory bodies (e.g., UNEP, German Federal Environment Agency) was conducted using ISI Web of Science and Google Scholar. The search was not limited by years or language of publication. The search was initially conducted in March 2020 and updated in September 2022. The following search terms were used.

#### For HBCDDs

All fields: [hexabromocyclododecane* OR HBCD*] AND [(human milk) or (breast milk)]. This search produced 168 results, which were evaluated for their appropriateness. The criteria for inclusion were that the studies provided lipid-standardized HBCDD levels in human milk (either isomer-specific HBCDD or ∑HBCDD) and included basic information on the study population (country of residence, sampling year). Of the initial 168 studies identified, 49 met the criteria and were used for further analysis (Figure S1, data sources listed in Table 1).

**Table 1:** Countries Used in This Study Grouped According to the United Nations Geoscheme

region	countries included			PBDEs	HBCDDs
Africa	Congo	South Africa	South Africa	7 studies^79−85^	5 studies^79−83^
	Cote d’Ivoire	Mauritius	Tanzania		
	Djibouti	Morocco	Togo		
	Egypt	Niger	Tunisia		
	Ethiopia	Nigeria	Uganda		
	Ghana	Senegal	Zambia		
	Kenya				
					
Asia	China	Japan	Syria	53 studies^18,81,86−136^	15 studies^81,89,95,132−135,137−144^
	Georgia	Macao	Taiwan		
	India	Philippines	Tajikistan		
	Indonesia	Russia^a^	Vietnam		
	Israel	South Korea			
					
Central and South America and Caribbean	Antigua and Barbuda	Chile	Peru	3 studies^73,81,145^	2 studies^73,81^
	Barbados	Haiti	Suriname		
	Brazil	Jamaica	Uruguay		
					
Europe	Belgium	Greece	Romania	61 studies^1,19,29,30,59,81,146−200^	25 studies^23,59,76,81,146−151,153−159,167,185,189,193,201−204^
	Bulgaria	Hungary	Russia^a^		
	Croatia	Ireland	Slovakia		
	Cyprus	Italy	Spain		
	Czechia	Lithuania	Sweden		
	Denmark	Luxembourg	Switzerland		
	Faroe Islands	Moldova	Turkey		
	Finland	Netherlands	UK		
	France	Norway	Ukraine		
	Germany	Poland			
					
North America	Canada	Mexico	USA	25 studies^17,51,70,77,81,205−222^	5 studies^51,77,78,81,83^
Oceania	Australia	Kiribati	Tonga	7 studies^223−228^	2 studies^81,226^
	Fiji	New Zealand	Tuvalu		

a General samples from Russia were included within the European category. When a study specified a geographic region that was within the Asian part of Russia, it was included in Asia.

#### For PBDEs

All fields: [polybrominated diphenyl ether* OR PBDE*] AND [(human milk) or (breast milk)]. This search identified 1204 results, which were then evaluated for their appropriateness. Only studies reporting individual PBDE congeners were included, and four congeners were selected as indicators due to their prevalence in literature: BDE-47, BDE-99, BDE-153, and BDE-209. Studies also had to include lipid-standardized concentrations for at least one of these congeners and include information on the study population (country of residence, sampling year). Of the initial 1204 studies, 158 met the criteria and were used for further analysis (Figure S1, data sources listed in Table 1).

All available data (either primary data reporting individual concentrations or all summary statistics) were extracted from the articles to a spreadsheet database. Data reported from pooled samples were treated as mean values.

### Data Set Standardization

Statistical evaluation was carried out by R software (version R 4.1.2). The data sources were separated by compound, sampling location, and/or date of sample collection. These records were aggregated by country and date of sampling, characterized either by mean or median value, minimum, 5th, 10th, 25th, 75th, 90th, and 95th quantile and maximum or any combination of these descriptive statistics. If all primary data/summary statistics within an aggregate record were taken during one year, the aggregated record was assigned to that year. In the other cases, the aggregated record was assigned to the middle point between the years of the oldest and the newest primary data/summary statistic.

For HBCDDs, 41 aggregated records included both α-HBCDD and the sum of α, β, and γ-isomers. These 41 records were used for estimating the contribution of α-HBCDD to the sums. This contribution was 94.0%, showing clearly that α-HBCDD dominates over the β and γ isomers. This 94.0% was then used to extrapolate α-HBCDD from records where only ∑HBCDDs were reported for the original data set, resulting in 260 aggregated records for α-HBCDD.

Primary FR data from five locations^76−78^ were used to establish that α-HBCDD and PBDE concentrations in breast milk have a general log-normal distribution of primary data within an aggregate data record. On the basis of the log-normal distribution assumption, the maximum likelihood estimation was used to apply a log-normal distribution for each aggregated sample and thus estimate its median value in cases where only other summary statistics were reported. In a few cases, the maximum likelihood estimate was not directly applicable since the only descriptive statistic characterizing the aggregated sample was an arithmetic mean. In such cases, a median standard deviation based on the rest of the samples was used (0.62 ng/g lipid weight; lw for α-HBCDD, 0.32 ng/g lw for BDE-47, 0.23 ng/g lw for BDE-99, 0.35 ng/g lw for BDE 153, and 50.15 ng/g lw for BDE-209) to derive quantiles describing the expected log-normal distribution. Finally, since there are no theoretical minimal and maximal values for the log-normal statistical distribution, the min and max values were considered as (1/*n*)th and (1–1/*n*)th quantiles for aggregated samples with known *n*; for aggregated samples with an unknown number of primary samples, *n* = 40 was used as it was the median value of studies where *n* was specified (Figure S2).

### Temporal Pattern Meta-Analysis

Data were grouped regionally following the United Nations geoscheme (Table 1). With the use of the aggregate data by region and sampling year, a weighted Theil-Sen trend analysis was conducted, assigning each aggregated sample a weight in the range of 0.1 to 1.0 for 10 to 100 primary samples and 1.0 for more than 100 primary samples. A weighted Mann-Kendall test was then used for assessing the trends’ significance.

### Breakpoint Analysis

An additional type of temporal pattern analysis was applied to European and Asian data sets, as these continents had the most complete records for both PBDEs and HBCDDs and are of particular interest given the early introduction of FR regulations. To evaluate whether this early introduction of restrictions led to a change in the FR time patterns over time, breakpoint analysis was applied to find a time point when the slope of the trend breaks,^229^ i.e., suggesting a change in the rate of change of a given FR concentration in human milk. The breakpoint analysis identifies the breakpoint by aiming for normally distributed residuals of both linear trends before and after the breakpoint, which indicates an optimal fit. The method searches for all possible breakpoints (in this case in increments of whole years only) and selects the breakpoint with the smallest sum of squares of residuals. Only significant results according to the difference of halving times before and after the breakpoint are considered.^229^

### Analysis of Geographic Patterns

Additional comparative statistical analyses were conducted using Graphpad Prism 8.0.2 using all studies after the year 2000. Data were grouped according to geographic region (Table 1). The concentrations from the different regions were compared using Kruskall-Wallis nonparametric ANOVA tests, and individual regions were compared with all others using Dunn’s multiple comparison tests. For geographic patterns, significance was set at *p* < 0.05.

### Limitations

The quality of the analytical work performed in individual studies was not evaluated, in favor of allowing for a greater breadth of data to be incorporated in the meta-analysis. All studies were published in peer-reviewed journals or as reports available from reputable national/international organizations, leading to the assumption of an acceptable level of data quality. Some reports (notably the UNEP/WHO data included in the Stockholm Convention GMP reports) do not include analytical information, although data are produced by recognized, accredited laboratories, and exclusion of this data would lead to a substantial loss in geographic coverage. Additionally, older studies reflecting very early analyses of PBDEs and HBCDDs may have more generous allowances in terms of QA/QC; however, it was important for the temporal analyses that these could be included. However, a consequence of this is that not all studies will meet the most stringent QA/QC standards.

While breastfeeding mothers present a relatively homogeneous population with respect to age and sex, some additional factors can impact breast milk concentrations of FRs, and these could not be incorporated into our meta-analysis, primarily because of inconsistent data across studies.

#### Representativeness

Data from breastmilk only reflects the chemical burden in the breast-feeding female population. The prevalence of breastfeeding mothers also varies by socioeconomic status and cultural background.^230^

#### Parity

Some studies have shown that parity is related to differences in levels of persistent compounds in breast milk. Primiparous mothers have been shown to have higher HBCDD concentrations than multiparous mothers,^132^ while studies focused on PBDEs have not found a relationship.^84,187,207,231^ As information on parity was not consistently recorded for all data sources, we did not include this confounder in our analyses and used all available data, regardless of parity.

#### Duration of Lactation and Sample Collection

The timing of breast milk collection within the lactation period varied widely, from 1 week to 10 months after birth, although the most typical was 3–8 weeks after birth, following the guidance of the WHO/UNEP breast milk surveys.^232^ Whether this impacts levels of PBDEs and HBCDDs in milk is unclear. Some studies have reported variability^166,207^ or significant decreases^233^ in PBDE levels in breast milk up to a year postpartum; however, Harrad and Abdallah^151^ reported no change in HBCDDs in milk over 12 months of lactation.

#### Maternal Age

This is often identified as a determinant of breast milk levels; however, this is directly related to the understanding of the persistence of these FRs in the body and temporal changes in exposures within a country.^234^ Older maternal age has been associated with higher PBDE levels in some studies in breast milk^129,204^ and sera,^235^ while others have found no association^187^ or an inverse association of lower levels in breast milk from older mothers.^84,207^ Similarly for HBCDDs, Fujii et al. identified age-dependency of γ-HBCDD in milk,^132^ but not other HBCDD isomers, while Drage et al.^235^ found no age-dependency in HBCDDs in sera.

#### Selection of Breast Milk as Biomonitoring Matrix

While breast milk has the advantage of being noninvasive and widely monitored, BDE-209 preferentially partitions to serum lipids rather than milk lipids,^69^ leading to proportionally lower levels in breast milk compared with exposures. However, while milk:serum partitioning can vary by congener/compound,^69^ the relative geographic patterns and temporal trends (1227 samples from 1973 to 2019) should be appropriately captured by either matrix, as milk and serum concentrations are typically well-correlated.^215^ The limited number of spatial and temporal studies conducted on certain compounds, such as BDE-209 and HBCDD in human breast milk is also a limitation for this study.

#### Uncertainties Regarding Partitioning and Half-Lives of PBDEs in Human Breast Milk

Very few studies have been conducted on the human biological distributions and half-lives of PBDE and HBCDDs. The existing evidence suggests higher persistence of BDE-153 in the human body^68,236,237^ and decreased partitioning to milk for higher molecular weight FRs.^69^ As a result, comparisons of concentrations across congeners would not necessarily reflect exposure trends; however, the bulk of our analysis relies on trends built individually for each congener and thus should not be impacted by the uncertainty in partitioning and half-lives.

### Temporal Patterns: Search Strategy and Selection Criteria

We supplemented our meta-analysis of temporal patterns of PBDE and HBCDD exposure with a comprehensive review of published time trends. A literature search was conducted using ISI Web of Science and Google Scholar, initially in March 2020, updated in September 2022. Search terms for the temporal trend studies were a combination of chemical-related terms (polybrominated diphenyl ether*, PBDE*, hexabromocyclododecane*, HBCD*), matrix-related (human, blood, serum, plasma, milk), and trend-related (time-trend* or temporal*).

For the temporal trend analysis of PBDEs, 268 data sources were identified, and the data were further examined to identify only studies that reported any of the four indicator PBDEs (−47, −99, −153, or −209), reflected the general population (not occupational exposure), reported basic biomonitoring parameters (e.g., geographic region, matrix, year of sample collection, number of samples), and had at least two time points with harmonized analyses (e.g., by the same laboratory). This resulted in 24 studies which were included in the overview of temporal trends (Table S2). For the temporal trend analysis of HBCDDs, the literature search identified 88 data sources, and the inclusion criteria (reported either α- or ∑HBCDD, not occupational exposure, reported basic biomonitoring parameters) led to the inclusion of 20 studies (Table S3). The trends from these studies were extracted, using the interpretation of the study authors to determine whether a trend is classified as increasing or decreasing over time, or whether no time trend is apparent.

## Results

### Spatial Patterns

Available data were not equally distributed across all geographic regions. Most studies were conducted in Europe and Asia, while Central and South America, North America, and Oceania had only limited data (Table 1). Very few studies on human biomonitoring of HBCDDs have been conducted in North America, which is surprising, considering that the region is known to have had stringent flame retardant regulations and historically high use of BFRs.^3^

**Figure 1:**
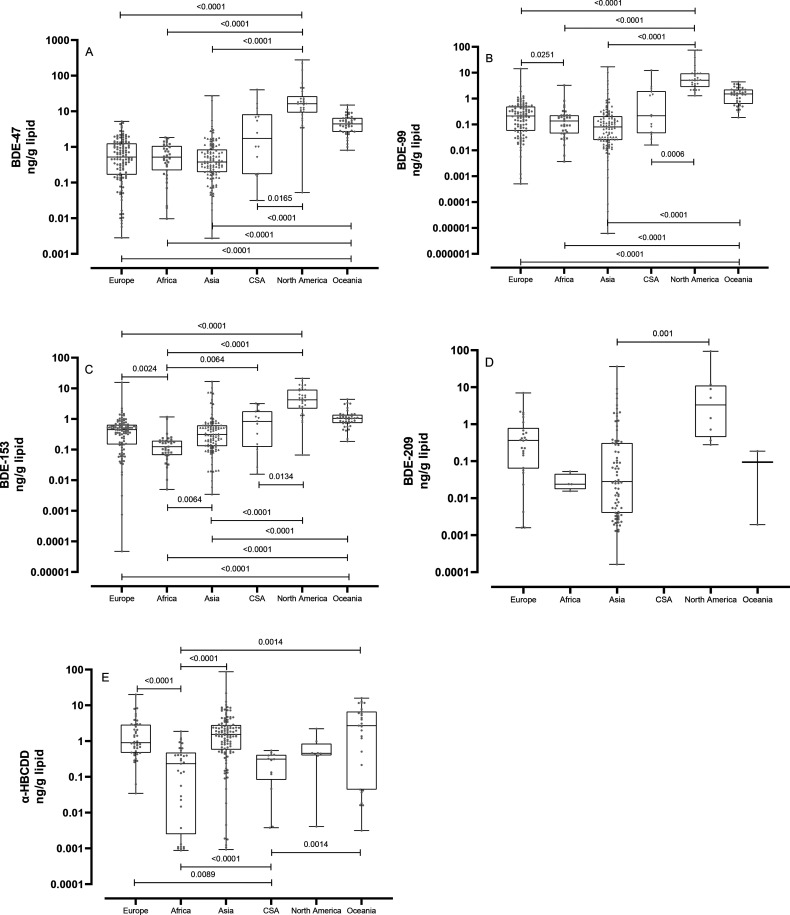
Box-and-whiskers (horizontal lines are medians, 95% confidence intervals, minima, and maxima) of (A) BDE-47, (B) BDE-99, (C) BDE-153, (D) BDE-209, and (E) α-HBCDD concentrations in different regions (CSA = Central and South America and the Caribbean), in breast milk data collected after year 2000. Nonparametric ANOVA tests (Kruskal–Wallis with Dunn’s post-tests) were conducted to determine significant differences.

Oceania had the highest median α-HBCDD concentration in breast milk (2.7 ng/g lipid), followed by Asia (1.5 ng/g lipid) and Europe (0.9 ng/g lipid) (Figure 1). The elevated concentrations of α-HBCDD in breast milk from Oceania were unexpected but may reflect a common market with many products from Asian manufacturers and Oceania implementing HBCDD regulations years later than Europe.^238^ Breast milk from Asia had significantly higher concentrations of α-HBCDD than milk from Africa, and Europe had significantly higher concentrations than Central and South America (Figure 1).

The concentrations of BDE-47, -99, and -153 in breast milk from North America were significantly higher than those from Europe, Africa, Asia, and Central- and South America: BDE-47, and -99 concentrations in North America were 39 and 65 times higher, respectively, than concentrations in Asia (Figure 1, Table S1). For the higher brominated compounds, North America had 50 and 138 times higher concentrations than Africa for BDE-153 and -209, respectively (Figure 1, Table S1). Europe had significantly higher concentrations of BDE-99 than Africa, and Africa had significantly lower concentrations of BDE-153 than all the other regions. For BDE-209, there were fewer data records which limited the comparison. The fact that BDE-209 is notoriously difficult to quantify due to high molecular mass and chemical instability likely contributes to the lack of data on levels in breast milk.^239,240^ For BDE-209, concentrations in milk from Asia were substantially lower than in North America, and no other regions had significant differences (Figure 1).

### Temporal Patterns

Africa, Oceania, and Central and South America had limited or no data on BDE-209 concentrations in breast milk, and temporal patterns could not be fully evaluated (Table 1; Figures S5, S7, and S8). The most prominent differences in trends are seen between Europe and North America for BDE-47, -153, and α-HBCDD. The temporal trends of BDE-99 and -209 are depicted elsewhere (Figures S3 and S4).

**Figure 2:**
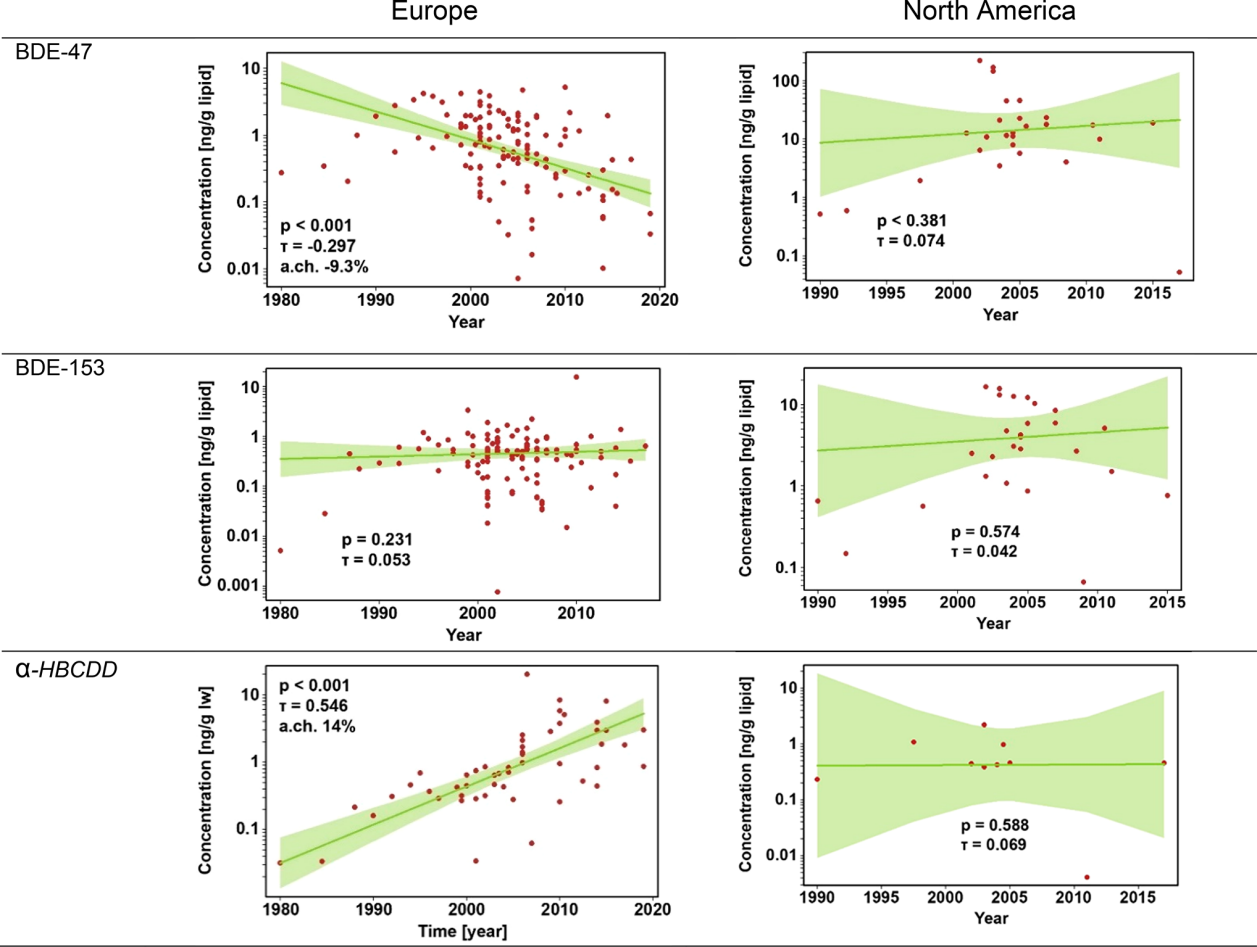
Weighted temporal trends of BDE-47, BDE-153, and α-HBCDD concentrations (ng/g lipid weight, lw) in breast milk from Europe and North America from literature. Shaded area indicates 95% confidence interval.

In Europe, BDE-47 and -99 decreased significantly (*p* = 0.0001; annual change of −9.3% and −10.1% respectively), while BDE-153 and -209 had no change over time (Figures 2 and S3). α-HBCDD concentrations increased significantly in the European population (*p* < 0.0001; 13.9% per annum) (Figure 2).

No significant changes (*p* < 0.05) were observed for any compound in North America (Figures 2 and S4), although we note that North American data was generally very sparse, limiting the ability to distinguish temporal trends. Notably, North America was the region with the least available α-HBCDD data points in breastmilk. The western hemisphere regions were the only regions where BDE-47 and -99 did not show a decreasing trend (Figures 2 and S4). The temporal patterns of all other geographical regions are presented in the Figures S5–S8.

Africa had a decrease in BDE-47 (*p* = 0.022; −7.8% per annum). Africa was the only continent where a significant decrease of α-HBCDD was observed (*p* < 0.0001; −33.6% per annum) (Figure S5). A similar pattern to Europe was seen in Asia. BDE-47 decreased (*p* = 0.002; −9.7 per annum), while BDE-153 and BDE-209 both increased, with BDE-209 increasing by 12.8% per annum (*p* = 0.05). α-HBCDD concentrations increased at a rate of 7.9% per annum (p = 0.006) (Figure S6).

In Central and South America and the Caribbean, BDE-153 increased significantly (*p* = 0.08) with an annual change of 14.8% (Figure S7). Oceania was the only region with a consistent decrease in all PBDE congeners. BDE-47 and -99 decreased significantly with an annual change of −13.1% and −15.5%, respectively. BDE-153 decreased at a rate of −6% per annum (*p* = 0.054) (Figure S8).

### Breakpoint Analysis

**Figure 3:**
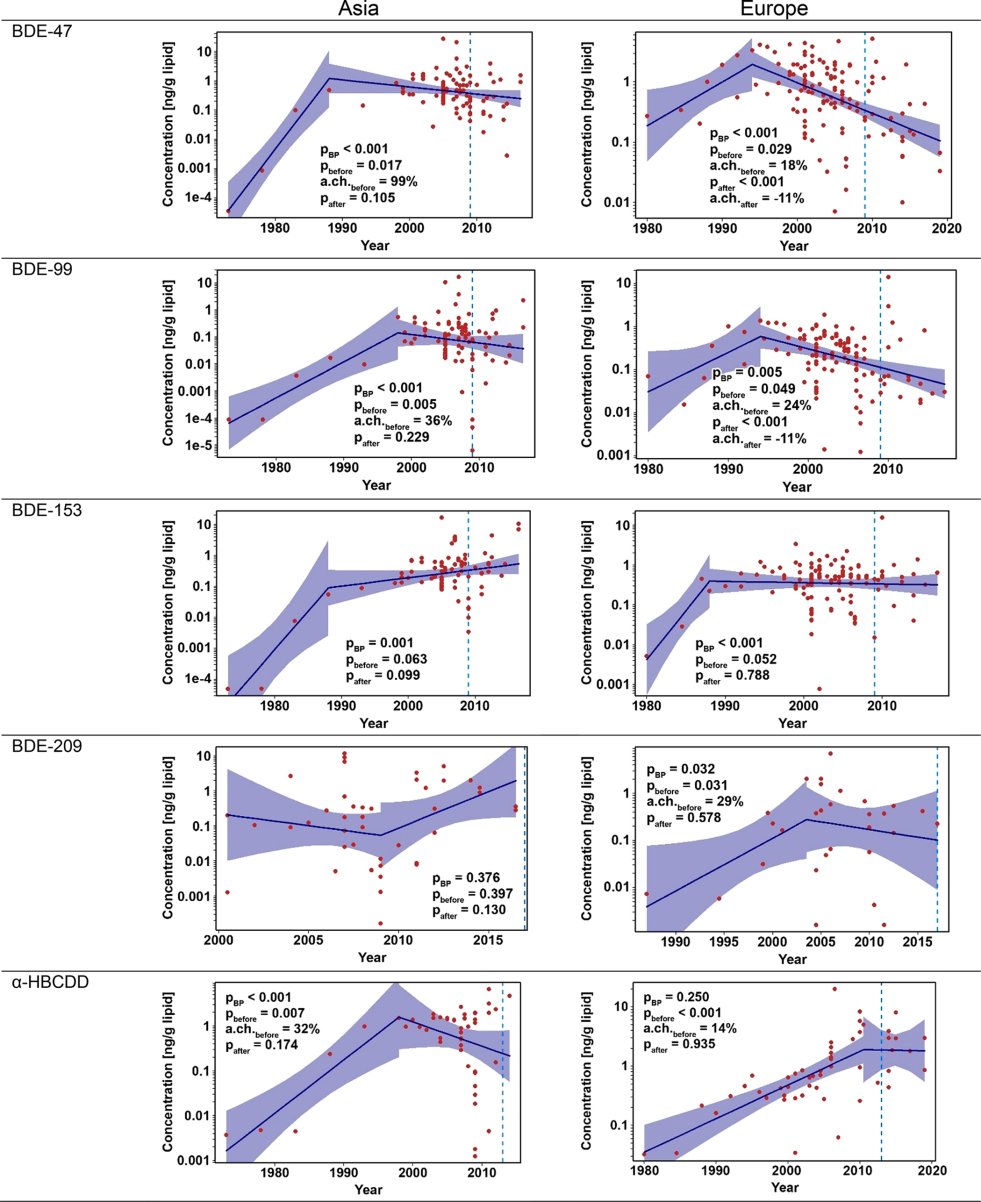
Results of breakpoint analysis for PBDEs in human milk for Asia and Europe for BDE-47, BDE-99, BDE-153, BDE-209, and α-HBCDD. The shaded area indicates the 95th percent confidence interval. The dotted blue line indicates when regulation was implemented by the Stockholm Convention (2009: BDE-47, -99, and -153; 2013: HBCDD; 2019: BDE-209). Please note that the Stockholm Convention date does not directly indicate the introduction of restrictions in each country; these may be earlier due to national/regional initiatives, or later, as Stockholm Convention parties enact regulations to implement the Convention. Thus, they are only indicative of the general timing of global restrictions.

Only Asia and Europe had sufficient data for breakpoint analyses (Figure 3). Breakpoints were calculated for other regions to identify the timing of concentration peaks (Figures S9–S12), but the trends are of limited value due to scarce data and are not discussed in detail.

In Europe, an increase in α*-*HBCDD of 13.9% per annum was observed from 1980 to 2010. In 2010, there was a change (breakpoint) in the temporal trends of α*-*HBCDD (Figure 3). The post-2010 decrease is not statistically significant due to limited data collected since 2010; thus, we cannot determine if recent concentrations are stable or declining. Although the breakpoint is not statistically significant, it represents the best fit for the available data and suggests a shift in exposure post-2010. In Asia, the α*-*HBCDD increase was even sharper (+31.6% per year; *p* = 0.007 Figure 3) and the breakpoint was identified earlier (1998), suggesting Asian concentrations reached a plateau at this point. Like Europe, the modeled decrease since the breakpoint in Asia is not statistically significant.

In both Asian and European breast milk data, the breakpoint in concentrations for BDE-47 and 99 was substantially earlier than for HBCDDs, close to 1990 for BDE-47 and between 1995 and 2000 for BDE-99. In all cases, concentrations decreased after the breakpoint for both congeners, but this postbreakpoint decrease is only significant for BDE-47 and BDE-99 in Europe (*p* > 0.0001).

However, there is a clear contrast between the breakpoints and before/after trends for BDE-153. The breakpoint for BDE-153 reflects only a change in the rate of increase of BDE-153 (Asia) or plateau (Europe) with no evidence of declining breast milk levels. Temporal patterns for BDE-209 in Asia were not significant, indicating no clear time trends. The European breakpoint for BDE-209 indicated a shift from the significant increase before 2004 to a current plateau or declining phase.

### Comparison with Other Reported Time Trends

**Figure 4:**
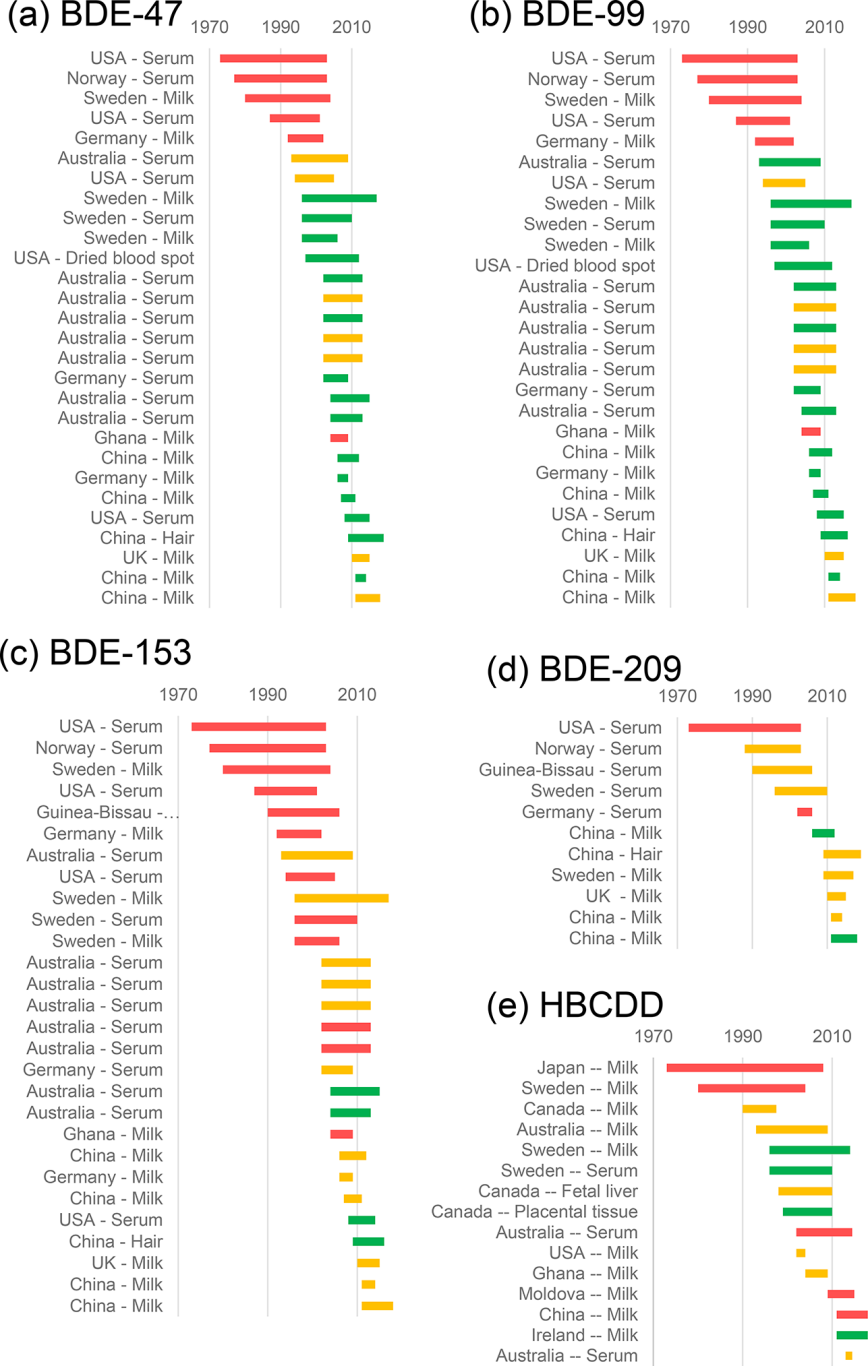
Time trends reported in human matrices in literature for (a) BDE-47, (b) BDE-99, (c) BDE-153, (d) BDE-209, and (e) HBCDD. Red bars indicate an increasing trend, yellow bars indicate no trend or a plateau, and green bars indicate a decreasing trend. Trends are classified based on the interpretations of the authors of each study. References for all studies can be found in Tables S2 and S3.

For the PBDEs, clear differences in the time trends by congener and by study timing are seen. For BDE-47 and -99 (Figure 4), there is a clear shift from early increasing time trends to more recent plateaus or decreasing time trends. The same pattern of early increasing trends and more recent reports of decreasing time trends is visible for BDE-153 (Figure 4), but the first decreases are not reported until much more recently. Most time trends for BDE-153 indicate a plateau. Relatively few studies report time trend analysis for HBCDD and BDE-209. For BDE-209 and HBCDD, no discernible time trend can be derived from literature for human matrices; trends were variable and not generalizable by region or duration of the time trend (Figure 4).

## Discussion and Implications

### Temporal Patterns

The analysis of published studies suggests that BDE-47 and -99 have a global decreasing trend. Decreasing temporal patterns were found in Europe, Asia, and Oceania. BDE-47 and -99 have human elimination half-lives of approximately 0.37–3 and 0.77–8 years, respectively.^68,236,237^ In the time since the penta- and octa-PBDEs were included in the Stockholm Convention in 2004, the population would have been exposed to lower concentrations of the compounds, and the existing compounds would have been eliminated from their bodies. The United States is a signatory to the Stockholm Convention but has yet to ratify or implement the convention in national legislation.^241^ The North American region was the only region with a continuous increase in BDE-47 and -99 (Figure 2).

While BDE-153 was also added to the Stockholm Convention in 2004, it does not share the same decreasing trends as the lower brominated congeners. All regions exhibited either a plateau or an increase in BDE-153 concentrations. Beyond the reduction in exposure due to the introduction of chemical restrictions, congener-specific differences in metabolism and storage in the body also affect breast milk trends differently. The human elimination half-life of BDE-153 is between 3.5 and 11.7 years.^68,236,237^ When considering worst case scenario, a half-life of up to 11.7 years would result in a 4-fold reduction (in the absence of continued exposure) of concentrations measured around 23 years ago, at the time of the implementation of the Stockholm Convention. Therefore, a full elimination would not be expected by the time of writing. This, coupled with continued exposure from existing products, could explain the lack of a decrease of BDE-153 in breast milk (Figures 2 and 4).

Global restrictions on penta- and octa-BDE technical mixtures, which are dominated by lower brominated congeners were generally between 2004 and 2013,^33,242,243^ whereas deca-BDE/BDE-209 was restricted only in 2017.^244^ Conclusions on BDE-209 are limited because of the lack of data and likely because the temporal changes are not yet significant enough to be identified in the generally short-time trend analyses that have been performed (Figure 4). The short half-life of BDE-209 in the body (e.g., 15 days in blood^245^), combined with the lack of a visible decline in BDE-209 levels in milk, suggests ongoing consistent BDE-209 exposures, despite recent restrictions in production, particularly in Asia, where the temporal trend (Figure S6) and the inverse breakpoint (Figure 3) indicated increasing concentrations in BDE-209 in breast milk. This agrees with our understanding of the recent high use of BDE-209 in consumer products and building materials on a global scale and the lag time between chemical restrictions and product replacements: emission of BDE-209 from in-use and waste stocks is estimated to continue until 2050.^3^

It is concerning how few studies investigated HBCDDs in residents from the Americas (*n* = 7), Africa (*n* = 5), and Oceania (*n* = 2) (Table 1). A similar problem was observed with PBDEs, where Africa (*n* = 7), Oceania (*n* = 7), and Central- and South America (*n* = 3) had limited studies, while Asia and Europe had more studies (*n* = 53 and 61, respectively) (Table 1). Without the proper information on the state of contamination in the Americas, particularly the highly developed and industrialized North America (*n* = 25 studies on PBDE), it is impossible to determine a global perspective on the state of human exposure.

Breakpoint analyses are useful tools to determine whether the accumulation trend of a compound has increased, plateaued, or decreased over time, highlighting the approximate time when the change occurred. In Asia and Europe, the broad time trends of FR concentrations in breast milk are closely tied to the timing of chemical restrictions (Figure 3). The European trend indicated a breakpoint in ∼2010 for α*-*HBCDD (Figure 3), which coincides with the increase in restrictions in Europe (identified as SVHC in 2009 and listed in Annex XIV of REACH in 2011)^40^ and provides evidence that the restrictions impacted HBCDD use and thus exposures in Europe. After 2010, it is unclear whether α-HBCDDs are in a plateau phase or whether we begin seeing evidence of a decrease in Europe and Asia (Figure 3), but it suggests ongoing human exposure at levels close to the European peak.

It is important to highlight that in Figure 3, none of the breakpoints observed for Europe or Asia align with the timing of the Stockholm Convention’s implementation (represented by the blue dotted line) but rather occurred earlier. This observation suggests that regional restrictions likely had a more pronounced impact than global restrictions. However, it should be acknowledged that the number of studies conducted after the implementation of the Stockholm Convention is limited compared to those conducted before its implementation, making it challenging to precisely assess the Convention’s effectiveness, especially concerning HBCDD and deca-BDE.

### Spatial Patterns

The strong contrast between PBDE concentrations in North America, particularly the USA, and most other regions is directly related to differences in flammability standards and PBDE use. BFRs have been quantified at higher concentrations in North America than in Europe in multiple matrices, including human tissue,^26^ house dust,^246^ and bird eggs.^247^ The USA has historically had higher concentrations of flame retardants in its consumer products compared with other regions due to stricter flammability standards.^248−250^ The fact that PBDE congeners still show an increasing time trend in North America (Figure 2) is likely linked to the large past use of PBDEs, combined with the later introduction of regulations. The United States has not ratified the Stockholm Convention and does not have any federal regulations on FR in existing uses, although 13 states have internal, state-wide concentration limits on FRs in selected products.^251^ Significant New Use Rules (SNURs), implemented by the United States Environmental Protection Agency in 2012, aim to ensure that any new uses of specific flame retardant chemicals undergo a thorough review and approval process prior to manufacturing or processing. The US EPA implemented SNURs for Penta and OctaBDE in 2006, ensuring no new use or manufacturing of these compounds.^252^ All manufacture, import, processing and distribution of decaBDE was under the US TSCA in 2021; however, significant exemptions remain, e.g., in motor vehicle parts until 2036.^253^ Large numbers of products containing PBDEs are likely still in use or circulation, which leads to continuous exposure and a higher body burden.

Surprisingly, breast milk from the Oceania region was also significantly higher than most other global regions, save North America (Figure 1). While most of the world reduced PBDE use in 2004, whether through regulation voluntary action, Australia only began implementing regulation on PBDE use, manufacturing, and import in 2007.^238^ Even though the import of PBDEs is banned, no regulation exists for the import of products that potentially could contain PBDEs, such as automobile parts, textiles, or electronic products.^254^

BDE-47 and -99, both primary components of penta-BDE, displayed similar spatial patterns (Figure 1), attributed to patterns in the use of technical penta-BDE formula worldwide.

### General Observations

A substantial lag-time exists between cessation of production and cessation of use of FRs because of the long half-lives of the compounds and the lifespan of products that they are used in. Significant reductions in production have a slower effect on use and emissions because of the large stock of PBDE-containing materials in use. Abbasi et al.^3^ estimated that the peak in PBDE use occurred in 2003; however, thousands of tons of PBDEs will remain in use in consumer products for decades. Plastic, textile, and electronic products containing FRs are still in use, to say nothing of buildings’ thermal insulation containing EPS or XPS, which accounts for more than 97% of the global HBCDD volume used.^255^ The ongoing human exposure to HBCDDs will be further mediated by HBCDD exposure through the renovation and demolition of buildings. Demolition activity can release significant amounts of building material-associated chemicals and demolition waste, including EPS or XPS panels, which are estimated to stay in place for ∼50 years before renovation takes place.^61^ Thus, direct exposures to HBCDDs in indoor spaces and environmental release will continue for many decades,^256^ effectively slowing the decreasing concentration levels through constant primary exposures. These can either contribute directly to either occupational or local population exposures, as well as increase the burden of secondary environmental exposures through landfill disposal and subsequent leaching of HBCDDs from the products.^31^

Furthermore, as we move toward a circular economy, there is significant potential for FRs to be incorporated into new consumer products made from recycled materials.^257,258^ Abbasi et al.^3^ estimated that 45000 t of PBDEs may reappear in new products made from recycled materials, such as plastics, food contact materials,^259,260^ and children’s toys.^261,262^ Due to the persistence of FRs, all environmental releases can contribute to secondary FR contamination in the surrounding air, soil, and water sources and lead to human exposure via dietary sources.^263^

The lack of recent biomonitoring studies on PBDEs and HBCDDs limits the evaluation of current population exposures and the impact of regulation on time trends. Of the human biomonitoring studies (Table 1 and Figure 4), less than 10% of the records cover the period after 2013, limiting our ability to evaluate the effectiveness of legislation on the global exposure trend of FRs. This may be due to the perception that once chemicals have been regulated, the problem has been dealt with, and it can be difficult to maintain interest and/or financial support for chemicals that are perceived to have been already addressed.^110^

Regulation has a quantifiable effect on the concentrations of FRs in human breast milk. Regions such as Asia and Europe where earlier regional regulations, prior to the Stockholm Convention, have been implemented had the clearest decline of most of the compounds that were considered in this study. Australia, where regulations were implemented later still shows elevated concentrations in human matrices, as does North America, which had the historically highest FR use globally. On top of use and regulation, the chemical characteristics of specific PBDE congeners also affect the response to regulation and can impact the time needed to evaluate the efficacy of policy actions. Although PBDEs and HBCDD are regulated through a ban on production, regulation will have different outcomes for different individual compounds based on their dominant use, the volume of historical use, and biological half-lives. However, it is encouraging to see that existing regulation and policy, when implemented early and comprehensively, had a positive impact on the decreasing human body burden of lower brominated flame retardants.

## Supporting Information Available

Figures showing temporal trend and breakpoint analysis for all flame retardants from all regions and tables detailing references used in literature review and meta-analysis (PDF)
