# Supplementary material for: Has Regulatory Action Reduced Human Exposure to Flame Retardants?
Source: Environ Sci Technol. 2023 Nov 22;57(48):19106–24. doi: 10.1021/acs.est.3c02896 (PMC10702444; doi:10.1021/acs.est.3c02896)
Supplement: Supplementary file 1 — es3c02896_si_001.pdf [file es3c02896_si_001.pdf]

Supplementary Information

Has regulatory action reduced human exposure to flame retardants?

Veronica van der Schyff[a], Jiří Kalina[a], Annalisa Abballe[b], Anna Laura Iamiceli[b], Eva Govarts[c], Lisa Melymuk[a,*]

[a] RECETOX, Faculty of Science, Masaryk University, Kotlarska 2, 611 37, Brno, Czech Republic

[b] Department of Environment and Health, Italian National Institute for Health, Viale Regina Elena 299, 00161 Rome, Italy

[c] VITO Health, Flemish Institute for Technological Research (VITO), 2400, Mol, Belgium

* Corresponding author: Lisa Melymuk, lisa.melymuk@recetox.muni.cz

List of Tables

Table S1 Comparisons of concentrations of flame retardants in breast milk from different regions after the year 2000. .... S3
Table S2 Previous studies evaluating time trends in PBDE concentrations in human biomonitoring matrices........ S4
Table S3 Previous studies evaluating time trends in HBCDD concentrations in human biomonitoring matrices. S5

List of Figures

Figure S1 Overview of data selection steps for literature review to identify reports of HBCDDs and PBDEs in human milk........ S6
Figure S2 Histogram of the number of samples included in studies with specific n reported S7
Figure S3. Weighted temporal trends of a) BDE-99, and b) BDE-209 concentrations in breast milk from Europe from literature. S8
Figure S4. Weighted temporal trends of a) BDE-99, and b) BDE-209 concentrations (ng/g lipid weight, lw) in breast milk from North America from literature S8
Figure S5. Weighted temporal trends of a) BDE-47, b) BDE-99, c) BDE-153, d) BDE-209, and e) α-HBCDD concentrations in breast milk from Africa from literature........ S9
Figure S6. Weighted temporal trends of a) BDE-47, b) BDE-99, c) BDE-153, d) BDE-209, and e) α-HBCDD concentrations in breast milk from Asia from literature. S10
Figure S7. Weighted temporal trends of a) BDE-47, b) BDE-99, c) BDE-153, d) α-HBCDD concentrations in breast milk from Central and South America and Caribbean from literature .S11
Figure S8. Weighted temporal trends of a) BDE-47, b) BDE-99, c) BDE-153, and d) α-HBCDD concentrations in breast milk from Oceania from literature S12
Figure S9. Results of breakpoint analysis for BDE-47 in human milk in (a) Central and South America and the Caribbean, (b) North America, and (c) Oceania S13

Figure S10. Results of breakpoint analysis for BDE-99 in human milk for (a) Central and South America and the Caribbean, (b) North America, and (c) Oceania S14
Figure S11. Results of breakpoint analysis for BDE-153 in human milk for (a) North America and (b) Oceania S14
Figure S12 Results of breakpoint analysis for BDE-209 in human milk for North America. S15

Table S1 Comparisons of concentrations of flame retardants in breast milk from different regions after the year 2000 in relation to other regions, calculated as the ratio of one region to another (e.g. North America has 19 times higher concentrations of BDE-47 compared with Europe).

BDE-47						
	Europe	Africa	Asia	CSA*	North America	Oceania
Europe	-	1.1	0.48	4	19	6.2
Africa	0.9	-	0.43	3.6	17	5.6
Asia	2.08	2.3	-	8.2	38	13
CSA*	0.25	0.12	0.12	-	4.7	1.6
North America	0.054	0.06	0.026	0.21	-	0.33
Oceania	0.16	0.18	0.078	0.64	3	-

BDE-99						
	Europe	Africa	Asia	CSA*	North America	Oceania
Europe	-	0.68	0.28	2.7	18	6.07
Africa	1.5	-	0.42	4	27	8.95
Asia	3.5	2.4	-	9.5	65	21.5
CSA*	0.37	0.25	0.11	-	6.8	2.3
North America	0.055	0.037	0.015	0.15	-	0.33
Oceania	0.16	0.11	0.046	0.44	3	-

BDE-153						
	Europe	Africa	Asia	CSA*	North America	Oceania
Europe	-	0.17	0.56	1.4	8.3	2.3
Africa	6	-	3.4	8.5	50	14
Asia	1.8	0.3	-	2.5	14	4.07
CSA*	0.71	0.12	0.4	-	5.9	1.6
North America	0.12	0.02	0.068	0.17	-	0.28
Oceania	0.44	0.073	0.25	0.62	3.64	-

BDE-209						
	Europe	Africa	Asia	CSA*	North America	Oceania
Europe	-	0.067	0.078	NA	9.2	0.26
Africa	15	-	1.17	NA	137	3.9
Asia	13	0.86	-	NA	117	3.4
CSA*	NA	NA	NA	NA	NA	NA
North America	0.11	0.0073	0.0085	NA	-	0.028
Oceania	3.8	0.26	0.3	NA	35	-

α-HBCDD						
	Europe	Africa	Asia	CSA*	North America	Oceania
Europe	-	0.26	1.7	0.34	0.5	3
Africa	3.9	-	6.5	1.4	2	12
Asia	0.6	0.15	-	0.21	0.3	1.8
CSA*	2.9	0.74	4.8	-	1.5	8.7
North America	2	0.51	3.3	0.69	-	6
Oceania	0.33	0.085	0.56	0.11	0.17	-

* CSA: Central and South America and the Caribbean

Table S2 Previous studies evaluating time trends in PBDE concentrations in human biomonitoring matrices.

Reference	Start	End	Span (years)	Trend				Continent	Country	Matrix
				BDE-47	BDE-99	BDE-153	BDE-209			
**Asante et al. 2011**[1]	2004	2009	5	Increase	Increase	Increase	NA*	Africa	Ghana	Milk
**Chen et al. 2019**[2]	2011	2014	3	Decrease	Decrease	Increase	NA	Asia	China	Milk
**Darnerud et al. 2015**[3]	1996	2010	14	Decrease	Decrease	Increase	No trend	Europe	Sweden	Serum
**Drage et al. 2019**[4]	2004	2015	11	Decrease	Decrease	Decrease	NA	Oceania	Australia	Serum
**Fängström et al. 2008**[5]	1980	2004	24	Decrease	Decrease	Increase	No trend	Europe	Sweden	Milk
**Fürst 2006**[6]	1992	2002	10	Increase	Increase	Increase	NA	Europe	Germany	Milk
**Gyllenhammar et al. 2021**[7]	1996	2017	21	Decrease	Decrease	No trend	No trend	Europe	Sweden	Milk
**Hoopmann et al. 2012**[8]	2006	2009	3	Decrease	Decrease	No trend	NA	Europe	Germany	Milk
**Lignell et al. 2009**[9]	1996	2006	10	Decrease	Decrease	Increase	NA	Europe	Sweden	Milk
**Linderholm et al. 2010**[10]	1990	2006	16	NA	NA	Increase	No trend	Africa	Guinea-Bissau	Serum
**Link et al. 2012**[11]	2002	2009	7	Decrease	Decrease	No trend	NA	Europe	Germany	Serum
**Ma et al. 2013**[12]	1997	2012	15	Decrease	Decrease	NA	NA	North America	USA	Dried blood spot
**Parry et al. 2018**[13]	2008	2014	6	Decrease	Decrease	Decrease	NA	North America	USA	Serum
**Schecter et al. 2005**[14]	1973	2003	30	Increase	Increase	Increase	Increase	North America	USA	Serum
**Sjödin et al. 2004**[15]	1987	2001	14	Increase	Increase	Increase	NA	North America	USA	Serum
**Tang et al. 2022**[16]	2009	2016	7	Decrease	Decrease	Decrease	No trend	Asia	China	Hair
**Tao et al. 2017**[17]	2010	2015	5	No trend	No trend	No trend	No trend	Europe	UK	Milk
**Thomsen et al. 2007**[18]	1977	2003	26	Increase	Increase	Increase	NA	Europe	Norway	Serum
**Toms et al. 2012**[19]	1993	2009	16	No trend	Decrease	No trend	NA	Oceania	Australia	Serum
**Toms et al. 2018**[20]	2002	2013	11	No trend / Decrease	No trend / Decrease	No trend / Increase	NA	Oceania	Australia	Serum
**Turyk et al. 2010**[21]	1994	2005	11	No trend	No trend	Increase	NA	North America	USA	Serum
**Zhang et al. 2017**[22]	2007	2011	4	Decrease	Decrease	No trend	NA	Asia	China	Milk
**Zhang et al. 2014**[23]	2006	2012	6	Decrease	Decrease	No trend	Decrease	Asia	China	Milk
**Zhao et al. 2021**[24]	2011	2018	7	No trend	No trend	No trend	Decrease	Asia	China	Milk

*Not analyzed

Table S3 Previous studies evaluating time trends in HBCDD concentrations in human biomonitoring matrices.

Reference	Start	End	Span (years)	Trend	Continent	Country	Matrix
Fuji et al. 2018 [25]<br>Kakimoto et al. 2008[26]	1973	2008	35	Increasing	Asia	Japan	Milk
Fangstrom et al. 2008 [5]	1980	2004	24	Increasing	Europe	Sweden	Milk
Ryan and Rawn 2014[27]	1990	1997	7.5	No trend	North America	Canada	Milk
Toms et al. 2012 [19]	1993	2009	16	No trend	Oceania	Australia	Milk
Lignell et al. 2015 [28]	1996	2014	18	Decreasing	Europe	Sweden	Milk
Darnerud et al. 2015 [3]	1996	2010	14	Decreasing	Europe	Sweden	Serum
Rawn et al. 2014[29]	1998	2010	12	No trend	North America	Canada	Fetal liver
Rawn et al. 2014 [29]	1999	2010	11	Decreasing	North America	Canada	Placental tissue
Drage et al. 2017[30], 2019 [4]	2002	2014	12.5	Increasing, plateau after 2012	Oceania	Australia	Serum
Ryan and Rawn, 2014 [27]	2002	2004	2	No trend	North America	USA	Milk
Asante et al. 2011 [1]	2004	2009	5	No trend	Africa	Ghana	Milk
Tirsina et al. 2017 [31]	2009	2015	6	Increasing	Europe	Moldova	Milk
Zhao and Shi, 2021[24],<br>Huang et al., 2020 [32]	2011	2018	7	Increasing	Asia	China	Milk
Wemken et al. 2020[33];<br>Pratt et al. 2013[34]	2011	2018	7	Decreasing	Europe	Ireland	Milk
Drage et al. 2017[30], 2019[4]	2013	2014	1.5	No trend	Oceania	Australia	Serum

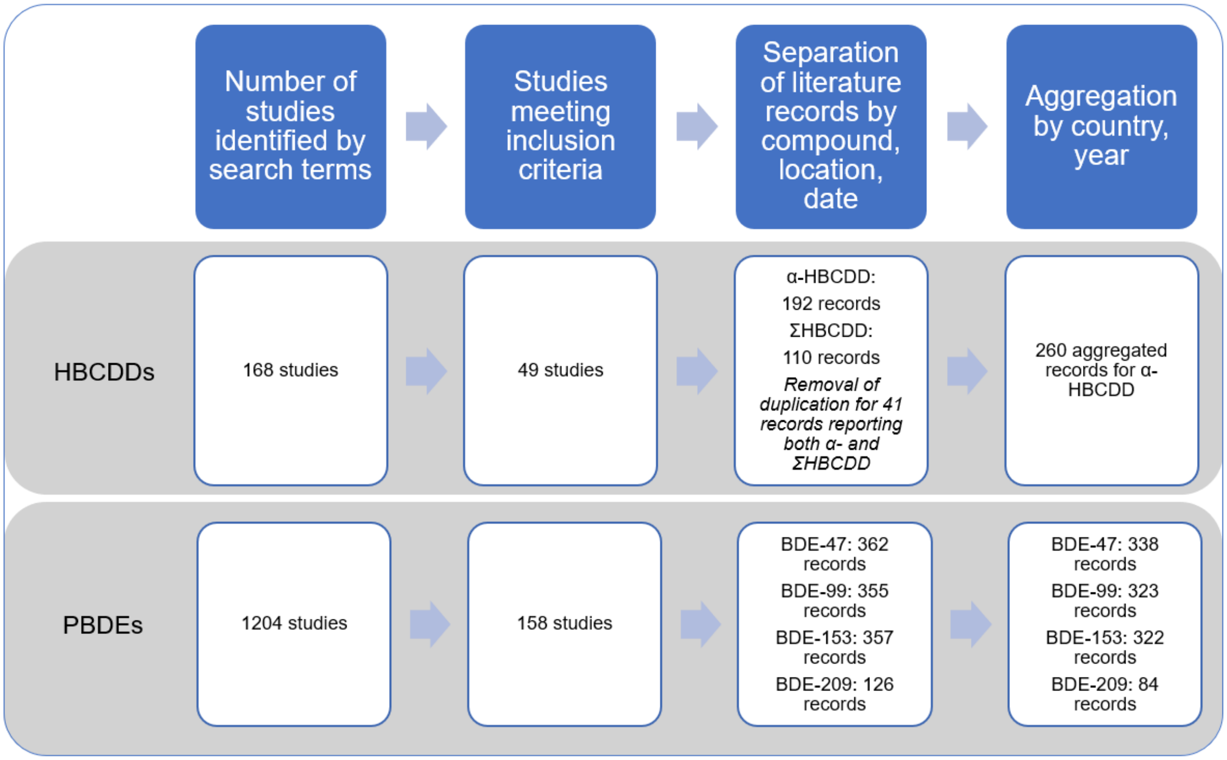

Figure S1. Overview of data selection steps for literature review to identify reports of HBCDDs and PBDEs in human milk

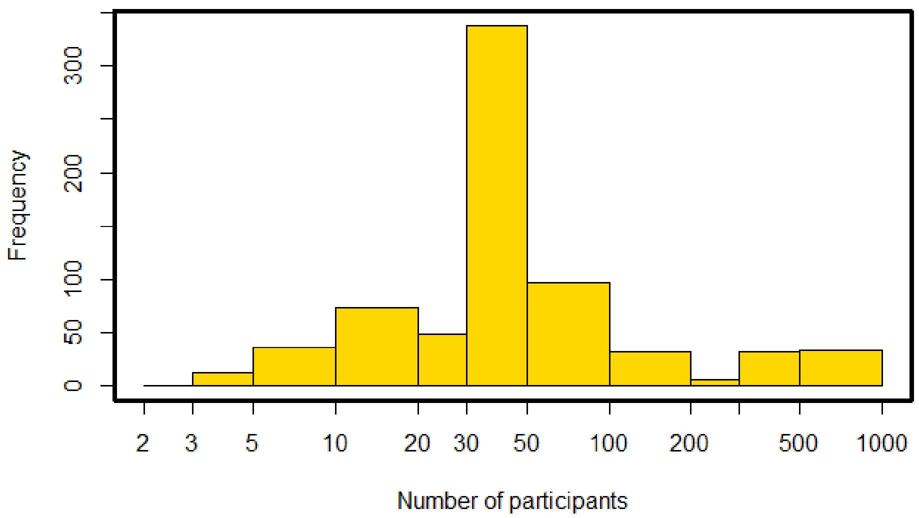

Figure S2. Histogram of the number of samples included in studies with specific n reported. The median was used to assign a sample number to studies where it was not specified.

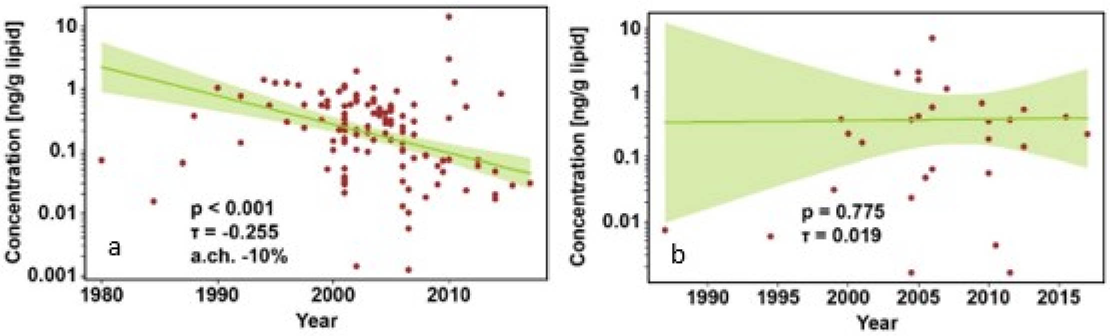

Figure S3. Weighted temporal trends of a) BDE-99, and b) *BDE-209 concentrations (ng/g lipid weight, lw) in breast milk from* Europe *from literature. Shaded area indicates 95th confidence internval.*

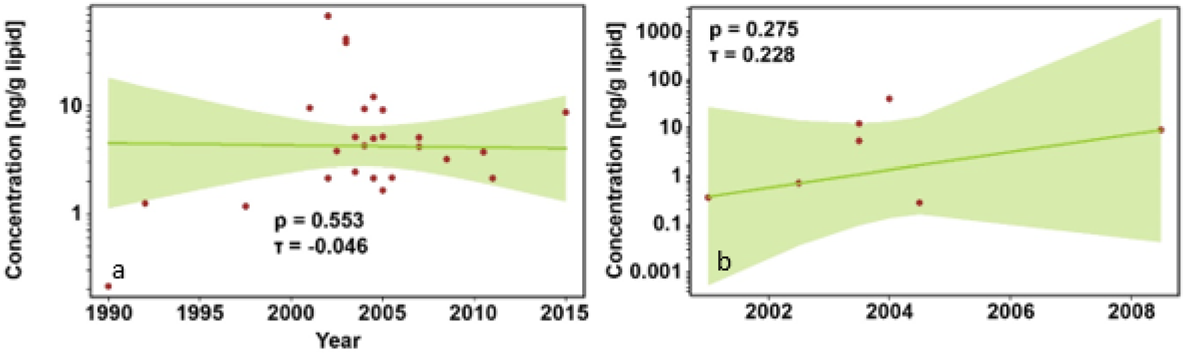

Figure S4. Weighted temporal trends of a) BDE-99, and b) *BDE-209 concentrations (ng/g lipid weight, lw) in breast milk from* North America *from literature. Shaded area indicates 95th confidence internval*

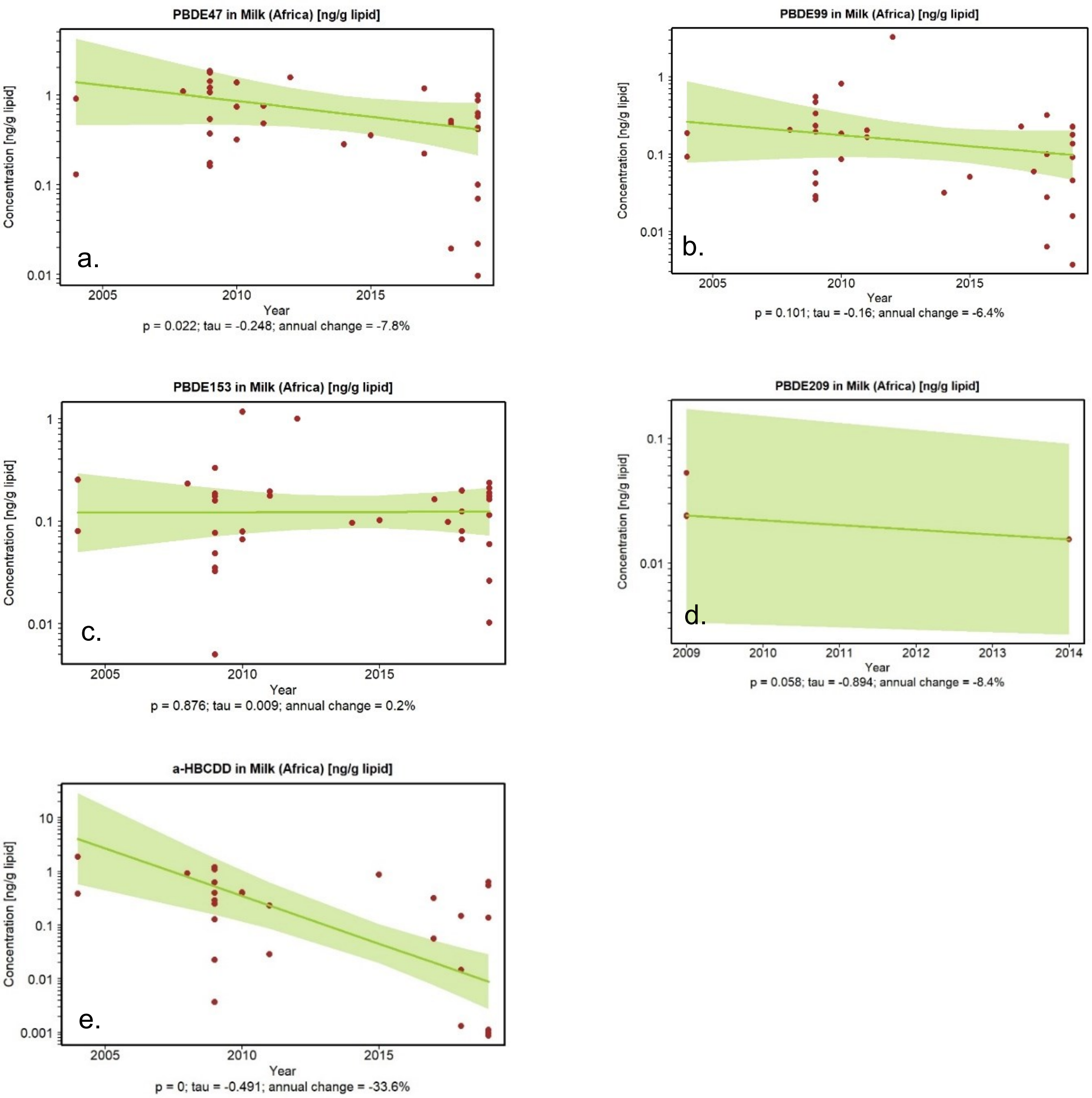

Figure S5. Weighted temporal trends of a) BDE-47, b) BDE-99, c) BDE-153, d) BDE-209, and e) α-HBCDD concentrations (ng/g lipid weight, lw) in breast milk from Africa from literature. Shaded area indicates 95th percent confidence interval

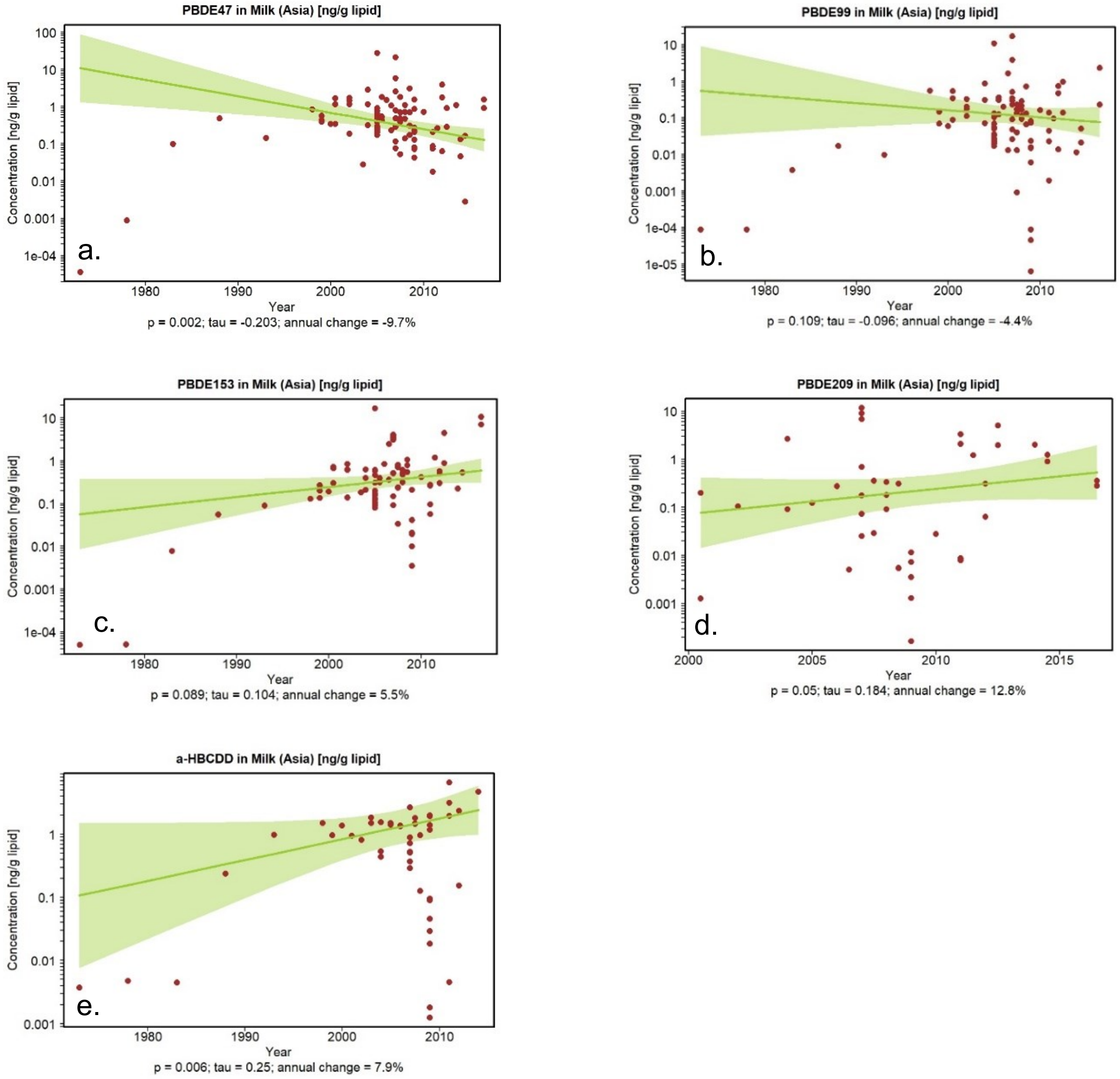

Figure S6. Weighted temporal trends of a) BDE-47, b) BDE-99, c) BDE-153, d) BDE-209, and e) α-HBCDD concentrations (ng/g lipid weight, lw) in breast milk from Asia from literature. Shaded area indicates 95th percent confidence interval.

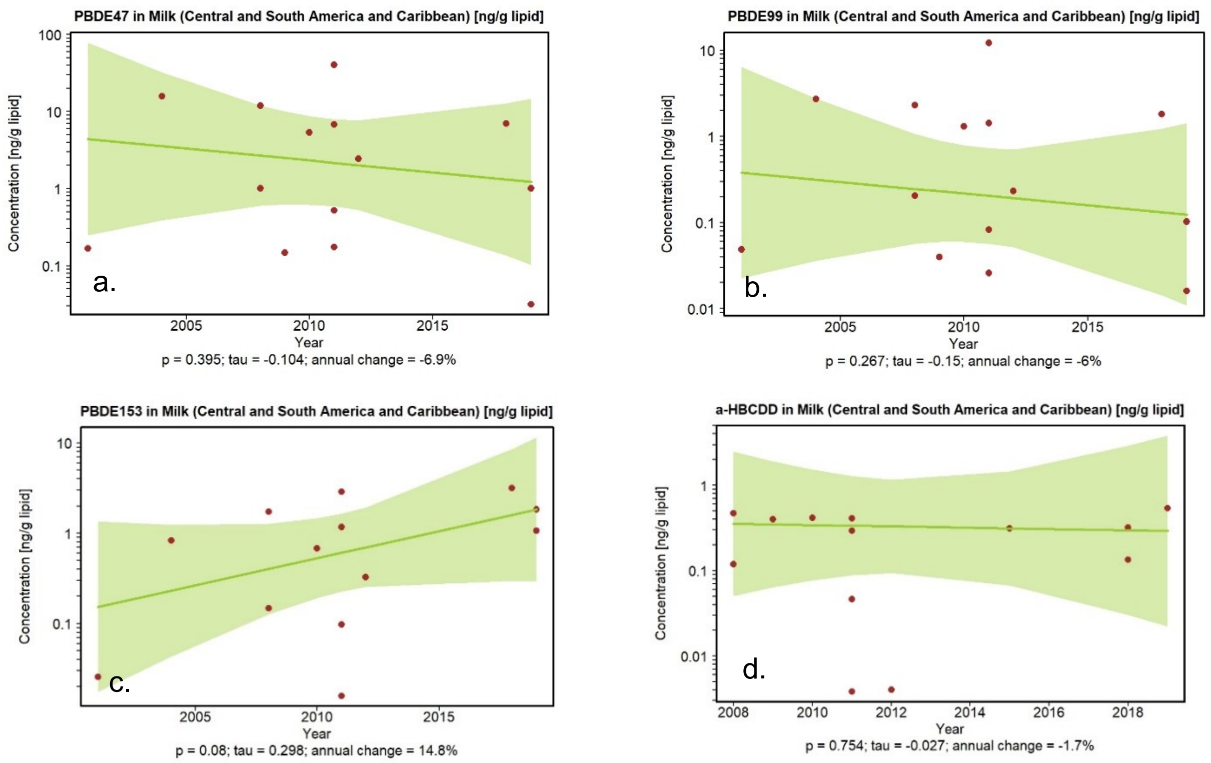

Figure S7. Weighted temporal trends of a) BDE-47, b) BDE-99, c) BDE-153, d) α-HBCDD concentrations (ng/g lipid weight, lw) in breast milk from Central and South America and the Caribbean from literature. Shaded area indicates 95th percent confidence interval

.

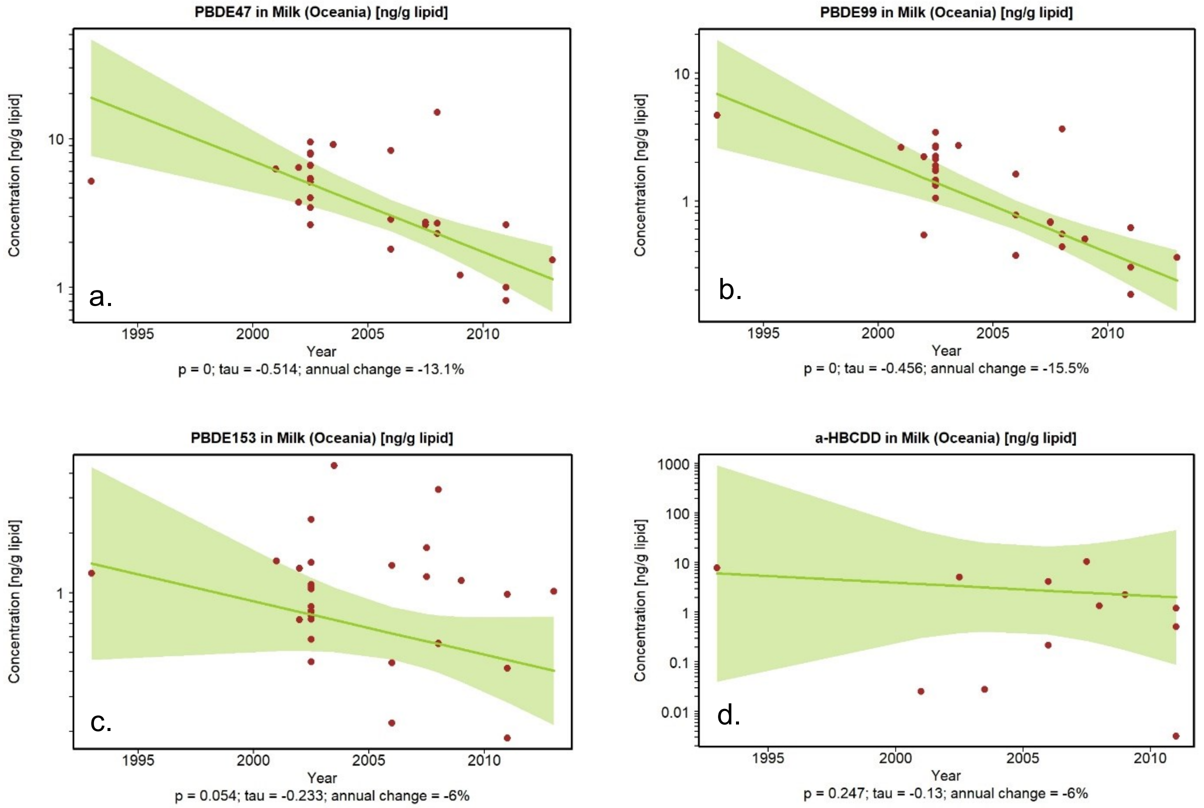

Figure S8. Weighted temporal trends of a) BDE-47, b) BDE-99, c) BDE-153, and d) α-HBCDD concentrations (ng/g lipid weight, lw) in breast milk from Oceania from literature. Shaded area indicates 95th percent confidence interval.

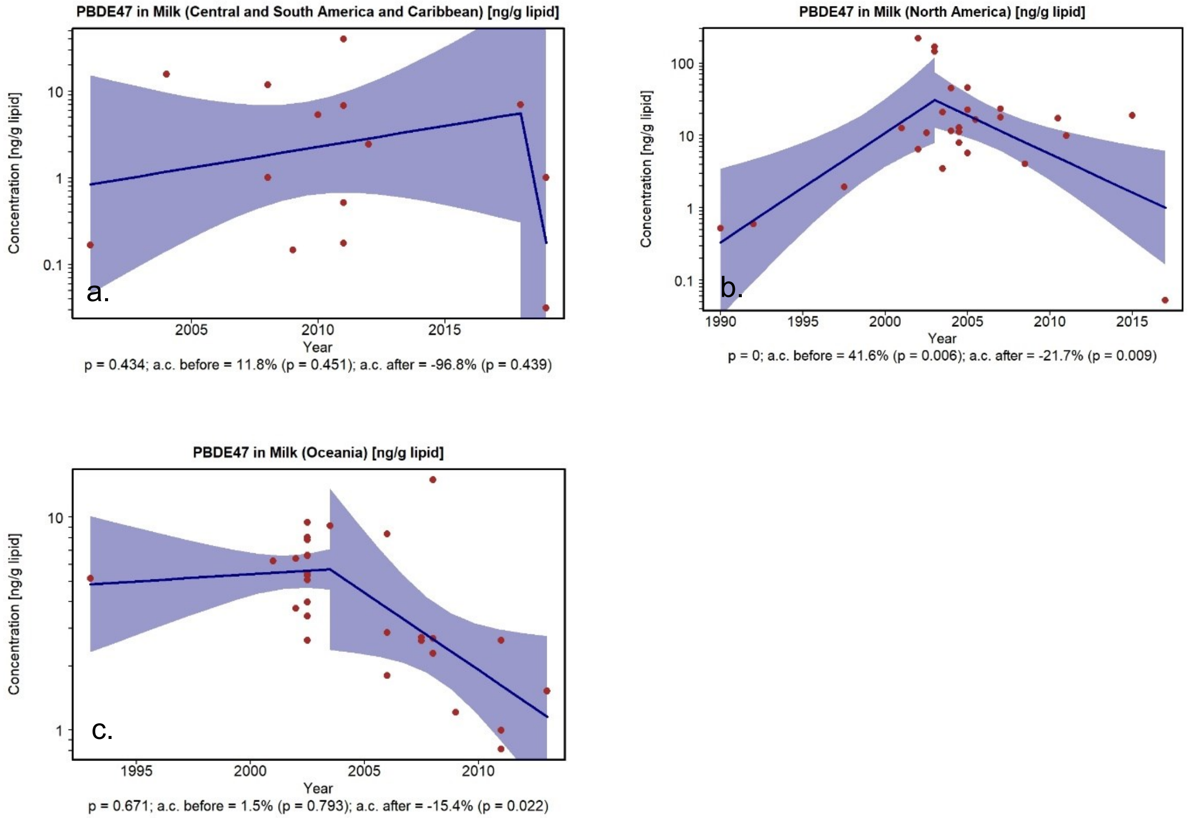

Figure S9. Results of breakpoint analysis for BDE-47 in human milk in (a) Central and South America and the Caribbean, (b) North America, and (c) Oceania. Shaded area indicates 95th percent confidence interval.

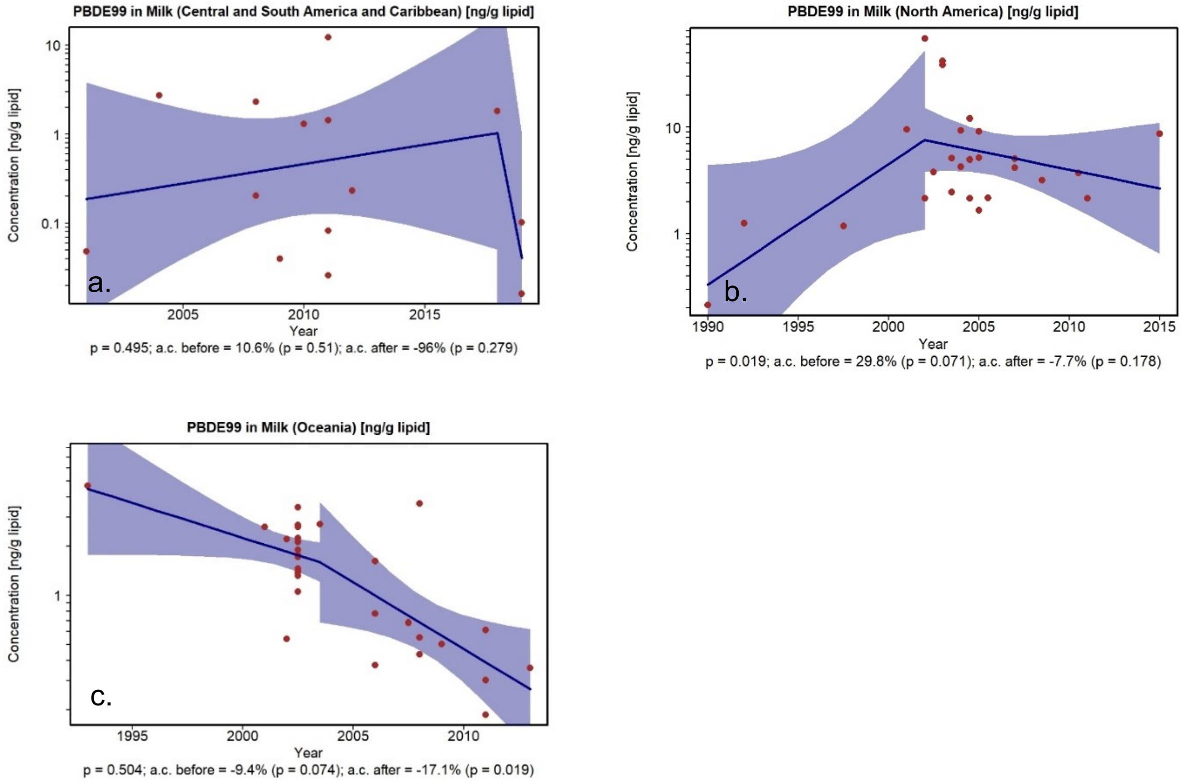

Figure S10. Results of breakpoint analysis for BDE-99 in human milk for (a) Central and South America and the Caribbean, (b) North America, and (c) Oceania. Shaded area indicates 95th percent confidence interval.

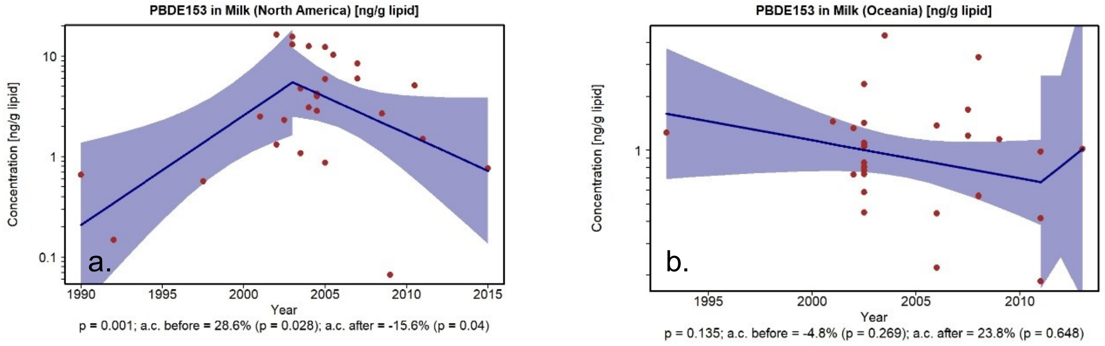

Figure S11. Results of breakpoint analysis for BDE-153 in human milk for (a) North America and (b) Oceania. Shaded area indicates 95th percent confidence interval.

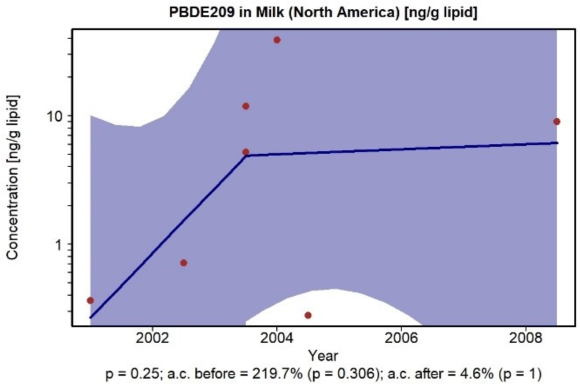

Figure S12 Results of breakpoint analysis for BDE-209 in human milk for North America. Shaded area indicates 95th percent confidence interval.

References

(1) Asante, K. A.; Adu-Kumi, S.; Nakahiro, K.; Takahashi, S.; Isobe, T.; Sudaryanto, A.; Devanathan, G.; Clarke, E.; Ansa-Asare, O. D.; Dapaah-Siakwan, S.; Tanabe, S. Human Exposure to PCBs, PBDEs and HBCDs in Ghana: Temporal Variation, Sources of Exposure and Estimation of Daily Intakes by Infants. *Environ Int* **2011**, *37* (5), 921–928. https://doi.org/10.1016/j.envint.2011.03.011.

(2) Chen, T.; Huang, M.; Li, J.; Li, J.; Shi, Z. Polybrominated Diphenyl Ethers and Novel Brominated Flame Retardants in Human Milk from the General Population in Beijing, China: Occurrence, Temporal Trends, Nursing Infants' Exposure and Risk Assessment. *Science of The Total Environment* **2019**, *689*, 278–286. https://doi.org/10.1016/J.SCITOTENV.2019.06.442.

(3) Darnerud, P. O.; Lignell, S.; Aune, M.; Isaksson, M.; Cantillana, T.; Redeby, J.; Glynn, A. Time Trends of Polybrominated Diphenylether (PBDE) Congeners in Serum of Swedish Mothers and Comparisons to Breast Milk Data. *Environ Res* **2015**, *138*, 352–360. https://doi.org/10.1016/j.envres.2015.02.031.

(4) Drage, D. S.; Harden, F. A.; Jeffery, T.; Mueller, J. F.; Hobson, P.; Toms, L. M. L. Human Biomonitoring in Australian Children: Brominated Flame Retardants Decrease from 2006 to 2015. *Environ Int* **2019**, *122*, 363–368. https://doi.org/10.1016/j.envint.2018.11.044.

(5) Fängström, B.; Athanassiadis, I.; Odsjö, T.; Norøn, K.; Bergman, Ške. Temporal Trends of Polybrominated Diphenyl Ethers and Hexabromocyclododecane in Milk from Stockholm Mothers, 1980-2004. *Mol. Nutr. Food Res* **2008**, *52*, 187–193. https://doi.org/10.1002/mnfr.200700182.

(6) Fürst, P. Dioxins, Polychlorinated Biphenyls and Other Organohalogen Compounds in Human Milk. *Mol Nutr Food Res* **2006**, *50* (10), 922–933. https://doi.org/10.1002/mnfr.200600008.

(7) Gyllenhammar, I.; Aune, M.; Fridén, U.; Cantillana, T.; Bignert, A.; Lignell, S.; Glynn, A. Are Temporal Trends of Some Persistent Organochlorine and Organobromine Compounds in Swedish Breast Milk Slowing Down? *Environ Res* **2021**, *197*. https://doi.org/10.1016/j.envres.2021.111117.

(8) Hoopmann, M.; Albrecht, U. V.; Gierden, E.; Huppmann, R.; Suchenwirth, R. Time Trends and Individual Characteristics Associated with Polybrominated Diphenyl Ethers in Breast Milk Samples 2006-2009 in Lower Saxony, Germany. *Int J Hyg Environ Health* **2012**, *215* (3), 352–359. https://doi.org/10.1016/j.ijheh.2011.08.019.

(9) Lignell, S.; Aune, M.; Darnerud, P. O.; Cnattingius, S.; Glynn, A. Persistent Organochlorine and Organobromine Compounds in Mother's Milk from Sweden 1996-2006: Compound-Specific Temporal Trends. *Environ Res* **2009**, *109* (6), 760–767. https://doi.org/10.1016/j.envres.2009.04.011.

(10) Linderholm, L.; Biague, A.; Månsson, F.; Norrgren, H.; Bergman, Å.; Jakobsson, K. Human Exposure to Persistent Organic Pollutants in West Africa - A Temporal Trend Study from Guinea-Bissau. *Environ Int* **2010**, *36* (7), 675–682. https://doi.org/10.1016/j.envint.2010.04.020.

(11) Link, B.; Gabrio, T.; Mann, V.; Schilling, B.; Maisner, V.; König, M.; Flicker-Klein, A.; Zöllner, I.; Fischer, G. Polybrominated Diphenyl Ethers (PBDE) in Blood of Children in Baden-

Württemberg between 2002/03 and 2008/09. *Int J Hyg Environ Health* **2012**, *215* (2), 224–228. https://doi.org/10.1016/j.ijheh.2011.10.018.

(12) Ma, W. L.; Yun, S.; Bell, E. M.; Druschel, C. M.; Caggana, M.; Aldous, K. M.; Buck Louis, G. M.; Kannan, K. Temporal Trends of Polybrominated Diphenyl Ethers (PBDEs) in the Blood of Newborns from New York State during 1997 through 2011: Analysis of Dried Blood Spots from the Newborn Screening Program. *Environ Sci Technol* **2013**, *47* (14), 8015–8021. https://doi.org/10.1021/es401857v.

(13) Parry, E.; Zota, A. R.; Park, J. S.; Woodruff, T. J. Polybrominated Diphenyl Ethers (PBDEs) and Hydroxylated PBDE Metabolites (OH-PBDEs): A Six-Year Temporal Trend in Northern California Pregnant Women. *Chemosphere* **2018**, *195*, 777–783. https://doi.org/10.1016/j.chemosphere.2017.12.065.

(14) Schecter, A.; Pa ̈pke, O.; Tung, K. C.; Joseph, J.; Harris, T. R.; Dahlgren, J. Polybrominated Diphenyl Ether Flame Retardants in the U.S. Population: Current Levels, Temporal Trends, and Comparison With Dioxins, Dibenzofurans, and Polychlorinated Biphenyls. *J Occup Environ Med* **2005**, *47* (3), 199–211.

(15) Sjödin, A.; Jones, R. S.; Focant, J. F.; Lapeza, C.; Wang, R. Y.; McGahee, E. E.; Zhang, Y.; Turner, W. E.; Slazyk, B.; Needham, L. L.; Patterson, D. G. Retrospective Time-Trend Study of Polybrominated Diphenyl Ether and Polybrominated and Polychlorinated Biphenyl Levels in Human Serum from the United States. *Environ Health Perspect* **2004**, *112* (6), 654–658. https://doi.org/10.1289/ehp.6826.

(16) Tang, B.; Chen, S. J.; Zheng, J.; Xiong, S. M.; Yan, X.; Luo, W. K.; Mai, B. X.; Yu, Y. J. Changes in Human Hair Levels of Organic Contaminants Reflecting China's Regulations on Electronic Waste Recycling. *Science of the Total Environment* **2022**, *806*. https://doi.org/10.1016/j.scitotenv.2021.150411.

(17) Tao, F.; Abou-Elwafa Abdallah, M.; Ashworth, D. C.; Douglas, P.; Toledano, M. B.; Harrad, S. Emerging and Legacy Flame Retardants in UK Human Milk and Food Suggest Slow Response to Restrictions on Use of PBDEs and HBCDD. *Environ Int* **2017**, *105*, 95–104. https://doi.org/10.1016/j.envint.2017.05.010.

(18) Thomsen, C.; Liane, V. H.; Becher, G. Automated Solid-Phase Extraction for the Determination of Polybrominated Diphenyl Ethers and Polychlorinated Biphenyls in Serum-Application on Archived Norwegian Samples from 1977 to 2003. *J Chromatogr B Analyt Technol Biomed Life Sci* **2007**, *846* (1–2), 252–263. https://doi.org/10.1016/j.jchromb.2006.09.011.

(19) Toms, L. M. L.; Guerra, P.; Eljarrat, E.; Barceló, D.; Harden, F. A.; Hobson, P.; Sjodin, A.; Ryan, E.; Mueller, J. F. Brominated Flame Retardants in the Australian Population: 1993-2009. *Chemosphere* **2012**, *89* (4), 398–403. https://doi.org/10.1016/j.chemosphere.2012.05.053.

(20) Toms, L. M. L.; Sjödin, A.; Hobson, P.; Harden, F. A.; Aylward, L. L.; Mueller, J. F. Temporal Trends in Serum Polybrominated Diphenyl Ether Concentrations in the Australian Population, 2002–2013. *Environ Int* **2018**, *121*, 357–364. https://doi.org/10.1016/j.envint.2018.09.014.

(21) Turyk, M. E.; Anderson, H. A.; Steenport, D.; Buelow, C.; Imm, P.; Knobeloch, L. Longitudinal Biomonitoring for Polybrominated Diphenyl Ethers (PBDEs) in Residents of

the Great Lakes Basin. *Chemosphere* **2010**, *81* (4), 517–522. https://doi.org/10.1016/j.chemosphere.2010.07.037.

(22) Zhang, L.; Yin, S.; Zhao, Y.; Shi, Z.; Li, J.; Wu, Y. Polybrominated Diphenyl Ethers and Indicator Polychlorinated Biphenyls in Human Milk from China under the Stockholm Convention. *Chemosphere* **2017**, *189*, 32–38. https://doi.org/10.1016/j.chemosphere.2017.09.014.

(23) Zhang, X.; Zhang, K.; Yang, D.; Ma, L.; Lei, B.; Zhang, X.; Zhou, J.; Fang, X.; Yu, Y. Polybrominated Biphenyl Ethers in Breast Milk and Infant Formula from Shanghai, China: Temporal Trends, Daily Intake, and Risk Assessment. *Science of the Total Environment* **2014**, *497*, 508–515. https://doi.org/10.1016/j.scitotenv.2014.08.034.

(24) Zhao, X.; Shi, Z. Legacy Brominated Flame Retardants in Human Milk from the General Population in Beijing, China: Biomonitoring, Temporal Trends from 2011 to 2018, and Nursing Infant's Exposure Assessment. *Chemosphere* **2021**, *285*. https://doi.org/10.1016/j.chemosphere.2021.131533.

(25) Fujii, Y.; Kato, Y.; Masuda, N.; Harada, K. H.; Koizumi, A.; Haraguchi, K. Contamination Trends and Factors Affecting the Transfer of Hexabromocyclododecane Diastereomers, Tetrabromobisphenol A, and 2,4,6-Tribromophenol to Breast Milk in Japan. *Environmental Pollution* **2018**, *237*, 936–943. https://doi.org/10.1016/j.envpol.2018.03.015.

(26) Kakimoto, K.; Akutsu, K.; Konishi, Y.; Tanaka, Y. Time Trend of Hexabromocyclododecane in the Breast Milk of Japanese Women. *Chemosphere* **2008**, *71* (6), 1110–1114. https://doi.org/10.1016/J.CHEMOSPHERE.2007.10.035.

(27) Ryan, J. J.; Rawn, D. F. K. The Brominated Flame Retardants, PBDEs and HBCD, in Canadian Human Milk Samples Collected from 1992 to 2005; Concentrations and Trends. *Environ Int* **2014**, *70*, 1–8. https://doi.org/10.1016/j.envint.2014.04.020.

(28) Lignell, S.; Aune, M.; Glynn, A.; Cantillana, T.; Fridén, U. *Report to the Swedish EPA (the Health-Related Environmental Monitoring Program)*; 2015.

(29) Rawn, D. F. K.; Gaertner, D. W.; Weber, D.; Curran, I. H. A.; Cooke, G. M.; Goodyer, C. G. Hexabromocyclododecane Concentrations in Canadian Human Fetal Liver and Placental Tissues. *Science of The Total Environment* **2014**, *468–469*, 622–629. https://doi.org/10.1016/J.SCITOTENV.2013.08.014.

(30) Drage, D. S.; Mueller, J. F.; Hobson, P.; Harden, F. A.; Toms, L. M. L. Demographic and Temporal Trends of Hexabromocyclododecanes (HBCDD) in an Australian Population. *Environ Res* **2017**, *152*, 192–198. https://doi.org/10.1016/J.ENVRES.2016.10.015.

(31) Tirsina, A.; Sircu, R.; Pinzaru, I.; Bahnarel, I. Changes over Time in Persistent Organic Pollutants (POP) Concentrations in Human Milk in the Republic of Moldova. *http://dx.doi.org/10.1080/02772248.2017.1287469* **2017**, *99* (5–6), 1007–1019. https://doi.org/10.1080/02772248.2017.1287469.

(32) Huang, M.; Li, J.; Xiao, Z.; Shi, Z. Tetrabromobisphenol A and Hexabromocyclododecane Isomers in Breast Milk from the General Population in Beijing, China: Contamination Levels, Temporal Trends, Nursing Infant's Daily Intake, and Risk Assessment. *Chemosphere* **2020**, *244*. https://doi.org/10.1016/j.chemosphere.2019.125524.

(33) Wemken, N.; Drage, D. S.; Cellarius, C.; Cleere, K.; Morrison, J. J.; Daly, S.; Abdallah, M. A. E.; Tlustos, C.; Harrad, S.; Coggins, M. A. Emerging and Legacy Brominated Flame

Retardants in the Breast Milk of First Time Irish Mothers Suggest Positive Response to Restrictions on Use of HBCDD and Penta- and Octa-BDE Formulations. *Environ Res* **2020**, *180*. https://doi.org/10.1016/j.envres.2019.108805.

(34) Pratt, I.; Anderson, W.; Crowley, D.; Daly, S.; Evans, R.; Fernandes, A.; Fitzgerald, M.; Geary, M.; Keane, D.; Morrison, J. J.; Reilly, A.; Tlustos, C. Brominated and Fluorinated Organic Pollutants in the Breast Milk of First-Time Irish Mothers: Is There a Relationship to Levels in Food? *Food Addit Contam Part A Chem Anal Control Expo Risk Assess* **2013**, *30* (10), 1788–1798. https://doi.org/10.1080/19440049.2013.822569.
